# Whole exome sequence analysis in 51 624 participants identifies novel genes and variants associated with refractive error and myopia

**DOI:** 10.1093/hmg/ddac004

**Published:** 2022-01-12

**Authors:** Jeremy A Guggenheim, Rosie Clark, Jiangtian Cui, Louise Terry, Karina Patasova, Annechien E G Haarman, Anthony M Musolf, Virginie J M Verhoeven, Caroline C W Klaver, Joan E Bailey-Wilson, Pirro G Hysi, Cathy Williams, CREAM Consortium, UK Biobank Eye Vision Consortium

**Affiliations:** School of Optometry & Vision Sciences, Cardiff University, Cardiff, CF24 4HQ, UK; School of Optometry & Vision Sciences, Cardiff University, Cardiff, CF24 4HQ, UK; School of Optometry & Vision Sciences, Cardiff University, Cardiff, CF24 4HQ, UK; School of Optometry & Vision Sciences, Cardiff University, Cardiff, CF24 4HQ, UK; Section of Ophthalmology, School of Life Course Sciences, King’s College London, WC2R 2LS, UK; Department of Twin Research and Genetic Epidemiology, School of Life Course Sciences, King’s College London, WC2R 2LS, UK; Department of Ophthalmology, Erasmus Medical Center GD, 3015GD Rotterdam, The Netherlands; Department of Epidemiology, Erasmus Medical Center GD, 3015GD Rotterdam, The Netherlands; Statistical Genetics Section, Computational and Statistical Genomics Branch, Nation Human Genome Research Institute, National Institutes of Health, Baltimore, MD 21224, USA; Department of Ophthalmology, Erasmus Medical Center GD, 3015GD Rotterdam, The Netherlands; Department of Clinical Genetics, Erasmus Medical Center GD, 3015GD Rotterdam, The Netherlands; Department of Ophthalmology, Erasmus Medical Center GD, 3015GD Rotterdam, The Netherlands; Department of Epidemiology, Erasmus Medical Center GD, 3015GD Rotterdam, The Netherlands; Department of Ophthalmology, Radboud University Medical Center, 6525EX Nijmegen, The Netherlands; Institute of Molecular and Clinical Ophthalmology Basel, CH-4031 Basel, Switzerland; Statistical Genetics Section, Computational and Statistical Genomics Branch, Nation Human Genome Research Institute, National Institutes of Health, Baltimore, MD 21224, USA; Section of Ophthalmology, School of Life Course Sciences, King’s College London, WC2R 2LS, UK; Department of Twin Research and Genetic Epidemiology, School of Life Course Sciences, King’s College London, WC2R 2LS, UK; Centre for Academic Child Health, Population Health Sciences, Bristol Medical School, University of Bristol, Bristol, BS8 1NU, UK

## Abstract

Refractive errors are associated with a range of pathological conditions, such as myopic maculopathy and glaucoma, and are highly heritable. Studies of missense and putative loss of function (pLOF) variants identified via whole exome sequencing (WES) offer the prospect of directly implicating potentially causative disease genes. We performed a genome-wide association study for refractive error in 51 624 unrelated adults, of European ancestry, aged 40–69 years from the UK and genotyped using WES. After testing 29 179 pLOF and 495 263 missense variants, 1 pLOF and 18 missense variants in 14 distinct genomic regions were taken forward for fine-mapping analysis. This yielded 19 putative causal variants of which 18 had a posterior inclusion probability >0.5. Of the 19 putative causal variants, 12 were novel discoveries. Specific variants were associated with a more myopic refractive error, while others were associated with a more hyperopic refractive error. Association with age of onset of spectacle wear (AOSW) was examined in an independent validation sample (38 100 early AOSW cases and 74 243 controls). Of 11 novel variants that could be tested, 8 (73%) showed evidence of association with AOSW status. This work identified *COL4A4* and *ATM* as novel candidate genes associated with refractive error. In addition, novel putative causal variants were identified in the genes *RASGEF1*, *ARMS2*, *BMP4*, *SIX6*, *GSDMA*, *GNGT2*, *ZNF652* and *CRX.* Despite these successes, the study also highlighted the limitations of community-based WES studies compared with high myopia case–control WES studies.

## Introduction

Myopia represents the negative arm and hyperopia represents the positive arm of the refractive error distribution. Myopia is characterized by excessive elongation of the eye during childhood, while hyperopic eyes typically remain shorter than average (1). The prevalence of myopia has increased in recent decades, especially in parts of East Asia, where the majority of young adults are now myopic (2). Myopia is a risk factor for glaucoma, cataract, retinal detachment and maculopathy, with the risk of retinal detachment and maculopathy increasing exponentially with each diopter (D) of myopia (3). Hyperopia is a risk factor for amblyopia and angle-closure glaucoma (4).

The rapid rise in the prevalence of myopia in East Asia strongly suggests a major role for environmental risk factors (5). However, refractive errors are highly heritable, and genome-wide association studies (GWASs) have identified >450 different regions of the human genome that confer susceptibility to refractive error (6,7). Consistent with this evidence for a role of both genetic and lifestyle factors, recent studies suggest that specific genetic variants act as risk factors for myopia in individuals who are exposed to a high-risk environment, such as intensive education, via gene–environment interaction (8,9). Monogenic forms of high myopia and high hyperopia have also been identified in which a high penetrance allele of large effect is present alongside the polygenic background (10,11).

Translating GWAS discoveries into new therapies to prevent myopia requires the identification of the genes and biological pathways through which the genetic risk variants exert their effects. In practice, it can be challenging to link a GWAS lead variant to the causal gene. Most mutations that give rise to monogenic disorders are predicted to have a direct functional effect on a specific protein, thus strongly implicating that protein in the disease process. In contrast, the majority of GWAS lead variants are not situated in exons and thus are not predicted to directly affect protein function; instead, they typically influence disease susceptibility by modifying the expression level of one or more nearby genes (12). The nearest gene to a GWAS lead variant can be assumed to be the causal gene, but this assumption may be incorrect, as genetic variants can influence the expression level of genes situated thousands of base-pairs upstream or downstream (13). Past GWAS analyses have only examined genetic variants that are relatively common in the population since rare genetic variants—defined as those with a minor allele frequency (MAF) of <1%—are difficult to characterize with conventional array-based genotyping and imputation. Here, we report the first large-scale GWAS for refractive error using whole exome sequencing (WES)-based genotyping, which provides high accuracy even for rare variants. The current GWAS was restricted to putative loss of function (pLOF) and missense variants, both of which have a high chance of altering protein function. This analysis provided new insight into the role of both common and rare coding variants in conferring susceptibility to myopia and hyperopia.

## Results

### GWAS for refractive error using WES data

Genotypes from the UK Biobank 200k WES release (October 2020) were tested for association with the spherical equivalent refractive error averaged between the right and left eyes of each participant [mean spherical equivalent refractive error averaged between fellow eyes (avMSE)] after applying a rank-inverse-normal transform (RINT) transformation (RINT-avMSE).

The majority of participants were recruited into the UK Biobank study in the period before ophthalmic assessments were introduced into the assessment schedule, which restricted the available sample size. In total, there were 51 624 unrelated individuals of European ancestry with exome sequence and autorefraction information available and who had no history of contraindicated eye disorders (Table 1). The median age of the sample was 59.0 years old [interquartile range (IQR): 12.5] and the median refractive error was +0.17 D (IQR: 2.33). Approximately, 53% of participants were female.

**Table 1:** Demographic characteristics of the GWAS participants; values are median (25–75th percentile)

Trait	All	Females	Males	*P*
	(*n* = 51 624)	(*n* = 27 612)	(*n* = 24 012)	
Age (years)	59.00 (51.46–63.92)	58.29 (51.08–63.42)	59.77 (52.00–64.50)	7.50E-46
avMSE (D)	+0.17 (−1.19 to +1.14)	+0.19 (−1.22 to +1.18)	+0.14 (−1.16 to +1.09)	6.70E-02
Age of completing education (years)	18.00 (16.00–21.00)	18.00 (16.00–21.00)	18.00 (16.00–21.00)	6.00E-03
Height (m)	1.69 (1.62–1.76)	1.63 (1.59–1.67)	1.76 (1.72–1.81)	<1.0E-99

We restricted attention to missense and pLOF variants with minor alleles occurring at least four times in the study sample. A total of 29 179 pLOF variants were tested of which 839 were common and 28 340 were rare. A total of 495 263 missense variants were tested of which 27 252 were common and 468 011 were rare. Quantile-quantile (QQ) plots suggested the evidence of association with the RINT-avMSE refractive error phenotype for common variants (MAF ≥ 0.01) in both annotation (Anno) categories (Supplementary Material, Fig. S1). In addition, missense variants in the MAF: 0.01–0.001 range also showed an excess of strong associations with refractive error. In contrast, there was no evidence for an above-chance level of association for ultra-rare variants (MAF < 0.001) in either of the Anno categories (Supplementary Material, Fig. S1). The Bonferroni method was applied to account for multiple testing of the 29 179 pLOF and 495 263 missense variants (524 442 variants in total). Accordingly, variants from the GWAS were taken forward for fine-mapping if *P* < 9.53E-08; where 9.53E-08 = 0.05/524442.

### pLOF variants associated with refractive error

**Figure 1:**
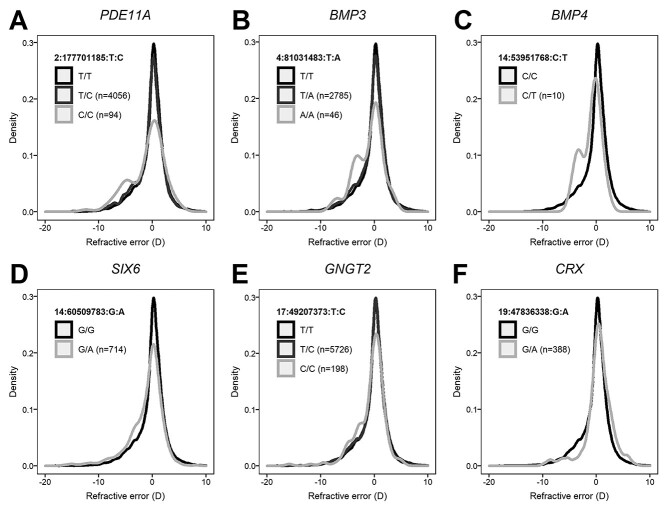
Distribution of refractive error in individuals carrying pLOF or missense variants in *PDE11A* (**A**), *BMP3* (**B**), *BMP4* (**C**), *SIX6* (**D**), *GNGT2* (**E**) and *CRX* (**F**). Note that certain risk alleles are associated with a more myopic refractive error, such as the ‘A’ allele of SIX6 variant 14:60509783:G:A, while other risk alleles are associated with a more hyperopic refractive error, such as the ‘A’ allele of CRX variant 19:47836338:G:A. A color version of this figure is available as Supplementary Material, Figure S3.

**Figure 2:**
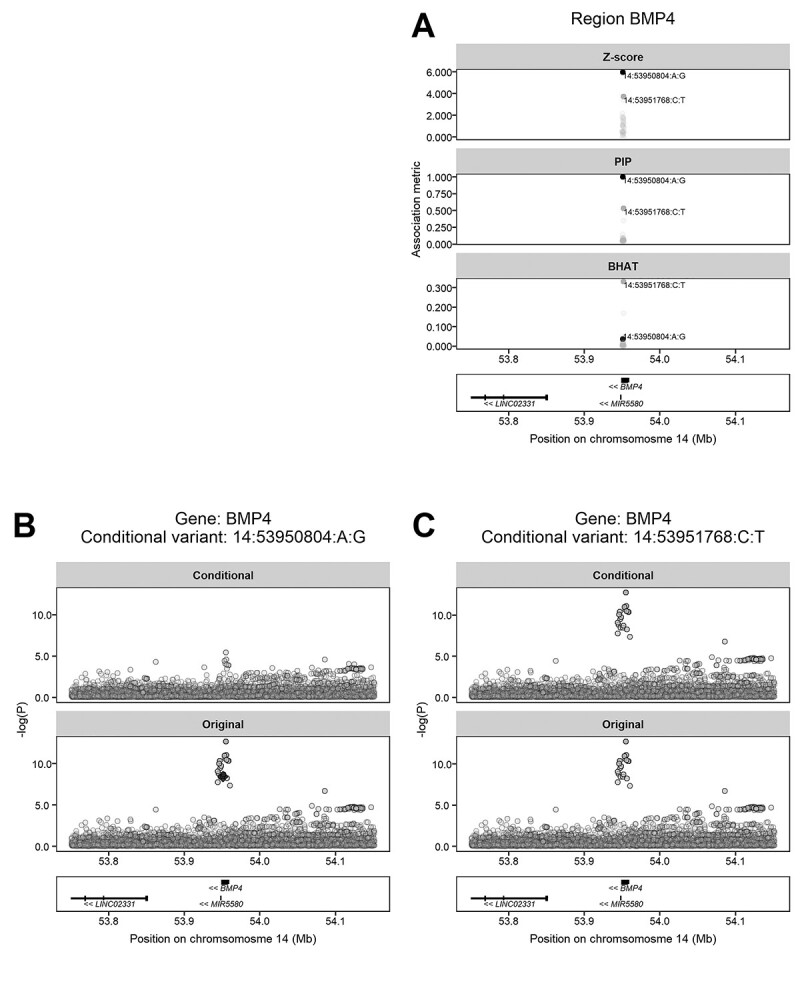
Fine-mapping and conditional analysis of the *BMP4* gene region. (**A**) Results from fine-mapping analysis with *SUSIE*. The images show the evidence for association with refractive error (‘*Z*-score’), the statistical confidence that a variant is a putative causal variant (‘PIP’; probability units on scale: 0–1), and the relative effect size of the variant (‘BHAT’; units of standard deviation change in refractive error per copy of risk allele). Independent putative causal variants are shaded more darkly. (**B** and **C**) Results of the conditional GWAS analyses. Images show GWAS results before (‘Original’) and after (‘Conditional’) conditioning on the specified lead variant in the region. The lead variant is depicted as a black diamond. Note that rare variant 14:53951768:C:T was not among the variants included in the original GWAS and that conditioning on this variant did not appreciably impact on the GWAS regional association plots (C). A color version of this figure is available as Supplementary Material, Figure S4.

Full details from the single marker GWAS analysis and the subsequent fine-mapping analyses are shown in Supplementary Material, Tables S1 and S2, respectively. Consistent with the QQ plots (Supplementary Material, Fig. S1), none of the 28 340 rare pLOF variants was associated with refractive error after correction for multiple testing. Furthermore, only 1 of the 839 pLOF variants with MAF ≥ 0.01 had evidence of association; this was a ‘stop lost’ variant in *BMP4* (14:53950804:A:G; *P* = 2.25E-09). Fine-mapping of the *BMP4* gene region with the *SUSIE* package (14) highlighted the pLOF mutation 14:53950804:A:G as a strong putative causal variant; fine-mapping posterior inclusion probability (PIP) = 1.00 (Fig. 2A). This variant, which is very common in the population (MAF = 0.42) but has a small effect size, was implicated in myopia development previously (15). Notably, however, fine-mapping identified a second, independently associated rare intronic variant in *BMP4* (14:53951768:C:T). The 10 individuals carrying this novel rare variant (MAF = 0.0002) had a refractive error that was −0.74 D more myopic, on average, than non-carriers (Table 3; Fig. 1C). A conditional GWAS analysis was performed in which imputed genotypes from array-based genotyping in the genomic region were tested for association with the RINT-avMSE refractive error phenotype before versus after conditioning on the genotype of the two putative causal variants (14:53950804:A:G and 14:53951768:C:T) identified in the fine-mapping analysis. The conditional analysis suggested the two putative causal variants in *BMP4* fully explained the GWAS signal in the region (Fig. 2B and C).

### Missense variants associated with refractive error

**Figure 3:**
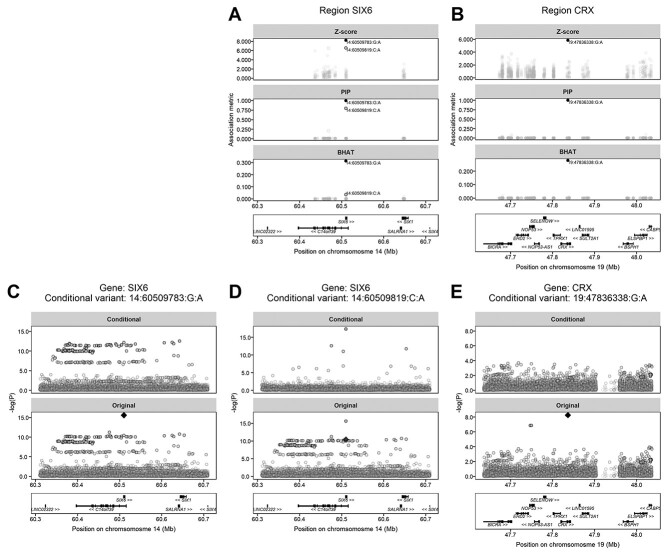
Fine-mapping and conditional analysis of the *SIX6* and *CRX* gene regions. (**A** and **B**). Results from fine-mapping analysis. (**C**–**E**) Results of the conditional GWAS analyses. See Figure 2 for details of images. A color version of this figure is available as Supplementary Material, Figure S5.

Two of the 468 011 rare missense variants were significantly associated with refractive error (Table 2). The first missense variant (14:60509783:G:A) introduces a p.Glu129Lys substitution in the *SIX6* gene. The second missense variant (19:47836338:G:A) introduces a p.Val66Ile substitution in the *CRX* gene. Both *SIX6* and *CRX* have previously been implicated in myopia development (7), however—as discussed later—the two novel, rare missense variants identified here had much larger effects than those identified in previous GWAS investigations (16). Individuals carrying a copy of the ‘A’ risk allele of the *SIX6* variant had a refractive error −0.87 D more negative, on average, compared with non-carriers, while individuals carrying a copy of the ‘A’ risk allele of the *CRX* variant had a refractive error +0.70 D more hyperopic, on average, compared with non-carriers (Table 3; Fig. 1D and F). Fine-mapping analysis of the genomic region surrounding each of the two novel rare missense variants provided evidence that both were putative causal variants: PIP = 1.000 and 0.999 for the *SIX6* and *CRX* variant, respectively (Fig. 3A and B; Supplementary Material, Table S2). Fine-mapping also suggested the presence of an additional novel missense variant in the *SIX6* gene (14:60509819:C:A) that was independently associated with refractive error after accounting for the effects of the original variant. The effect size of the second missense variant in *SIX6* was −0.12 D, which is more typical of effect sizes discovered in prior GWAS analyses for refractive error. Conditional analyses demonstrated that the two novel missense variants in the *SIX6* gene fully accounted for the GWAS signal in the region, as did the single novel missense variant in the *CRX* gene (Fig. 3C–E).

**Table 2:** Rare missense WES variants identified in GWAS for refractive error

Category	Gene	Variant	rsID	Chr	Pos	REF	ALT	EA	MAF	Beta	Standard error	*P*	Anno
Rare	*SIX6*	14:60509783:G:A	rs146737847	14	60509783	G	A	A	0.007	−0.302	0.037	1.68E-16	Missense
Rare	*CRX*	19:47836338:G:A	rs61748438	19	47836 338	G	A	A	0.004	0.288	0.049	4.92E-09	Missense

Abbreviations: Variant = gnomAD format identifier (Chromosome:Position:REF:ALT); REF = Reference allele present in GRCh38; ALT = alternate allele (this is the minor allele for the two variants listed here); rsID = dbSNP reference ID; Chr = chromosome; Pos = genomic position in GRCh38 coordinates; EA = effect allele; Beta = regression coefficient for RINT-avMSE per copy of the effect allele; *P* = *P*-value.

**Table 3:** Refractive error in individuals carrying risk alleles for pLOF or missense variants in *PDE11A, BMP3, BMP4, SIX6, GNGT2* and *CRX*; values are the average refractive error; only genotypes present in at least four individuals are included

Gene	Variant	rsID	MAF	Number of participants			Refractive error (D) (95% confidence interval)		
				REF/REF	REF/ALT	ALT/ALT	REF/REF	REF/ALT	ALT/ALT
*PDE11A*	2:177701185:T:C	rs17400325	0.041	47 447	4056	94	−0.23 (−0.26 to −0.21)	−0.52 (−0.61 to −0.44)	−1.00 (−1.67 to −0.33)
*BMP3*	4:81031483:T:A	rs74764079	0.028	48 792	2785	46	−0.24 (−0.26 to −0.22)	−0.53 (−0.63 to −0.42)	−1.05 (−1.82 to −0.28)
*BMP4*	14:53951768:C:T	rs534912805	0.0002	51 611	10	—	−0.26 (−0.28 to −0.23)	−1.00 (−2.29 to +0.30)	—
*SIX6*	14:60509783:G:A	rs146737847	0.007	50 909	714	—	−0.25 (−0.27 to −0.22)	−1.13 (−1.36 to −0.89)	—
*GNGT2*	17:49207373:T:C	rs35638197	0.059	45 696	5726	198	−0.24 (−0.26 to −0.21)	−0.40 (−0.47 to −0.33)	−0.70 (−1.09 to −0.31)
*CRX*	19:47836338:G:A	rs61748438	0.004	51 234	388	—	−0.26 (−0.29 to −0.24)	+0.44 (+0.17 to +0.70)	—

Abbreviations: Variant = gnomAD format identifier (Chromosome:Position:REF:ALT); REF = reference allele present in GRCh38; ALT = alternate allele (this is the minor allele for the six variants listed here); REF/REF = participants carrying zero copies of the rare allele; REF/ALT = participants carrying one copy of the rare allele; ALT/ALT = participants carrying two copies of the rare allele.

Analysis of the 27 252 missense variants with MAF > 0.01 led to the identification of 16 significantly associated variants, which were located in 12 distinct regions (Supplementary Material, Table S1). Fine-mapping these regions identified putative causal variants in the genes *PDE11A, COL4A4, PRSS56, BMP3, RASGEF1B, RGR, KAZALD1, ARMS2, ATM, GNB3, GSDMA, GNGT2* and *ZNF652* (Supplementary Material, Figs S6–S11). For seven of these genes (*COL4A4*, *RASGEF1B*, *ARMS2*, *ATM*, *GSDMA*, *GNGT2* and *ZNF652*) the putative causal variant was a novel discovery (Supplementary Material, Table S2), while for others, such as *PDE11A* variant 2:177701185:T:C (MAF = 0.04; Fig. 1A), the findings supported previous GWAS results. Notably, the novel variants included a missense variant in the *COL4A4* gene (2:227089883:G:A; MAF = 0.50). This variant was robustly associated with refractive error in the current GWAS (*P* = 1.06E-08) and strongly implicated as the putative causal variant in the region (PIP = 0.96); yet this gene has not previously been linked to myopia susceptibility to our knowledge. Conditional analyses suggested that the novel missense *COL4A4* gene variant accounted for the GWAS signal in the region (Supplementary Material, Fig. S6).

The discovery of novel missense variant 17:49207373:T:C (MAF = 0.06) in *GNGT2* exemplified the advantages of our combined WES plus fine-mapping approach. This variant was strongly associated with refractive error in the current WES GWAS (*P* = 3.91E-08), but fine-mapping suggested its association signal was partly driven by tagging a nearby variant (17:49312652:G:C) in *ZNF652* such that the combination of the two putative causal variants generated the very high GWAS signal (Supplementary Material, Fig. S8). The challenges of statistical fine-mapping were exemplified by the novel discovery of the *ATM* gene as the possible myopia susceptibility gene. In the *NPAT-ATM* region, which has not previously been implicated in myopia, fine-mapping analysis was unable to identify a putative causal variant definitively (Supplementary Material, Fig. S7). The lead WES GWAS variant in the vicinity, 11:108258930:A:G (*P* = 1.20E-08), had a fine-mapping PIP = 0.439. However, five other variants in the region had similar evidence of association, effect size and a PIP above the background level (red symbols in Supplementary Material, Fig. S7B), most likely owing to the high level of LD in the region.

### Validation: association with age of onset of spectacle wear

An independent sample of WES-genotyped participants with information available regarding their self-reported age of onset of spectacle wear (AOSW), and who were unrelated to any person in the WES GWAS sample, was selected as a ‘validation sample’. Individuals in the validation sample were classified as ‘likely myopia’ cases if they had an *AOSW* between 6 and 25 years of age. Participants not meeting this *AOSW* criterion were classified as controls. There were 38 100 cases and 74 243 controls. Demographic characteristics of the validation sample are presented in Supplementary Material, Table S3. Of the 19 putative causal variants identified in the fine-mapping analysis, 1 variant (14:53951768:C:T in *BMP4*) occurred too infrequently in the validation sample to provide a valid test of association. As shown in Table 4 and Supplementary Material, Table S4, a total of 9 out of the remaining 18 variants displayed evidence of an association with *AOSW* case–control status after accounting for multiple testing (*P* < 0.0028; *P*-value threshold: 0.05/18 = 0.0028), corresponding to a validation rate of 50%. Moreover, 14 out of 18 variants displayed at least nominal evidence of association (*P* < 0.05) with *AOSW* case–control status, giving a nominal validation rate of 78%. The direction of effect in the WES GWAS for refractive error was concordant with that in the *AOSW* case–control validation analysis for 17 of the 18 putative causal variants (95% concordance rate for direction of effect; Table 4). All of the variants showing evidence of association in the validation analysis were concordant in their direction of effect.

**Table 4:** Testing for independent validation of genetic association; the lead fine-mapped WES variants were tested for association with self-reported AOSW in an independent sample of *n* = 38 100 cases and *n* = 74 243 controls; cases were defined as having an AOSW between 6 and 25 years old

Gene	Variant	Number of controls			Number of cases			*P*	Validation direction	GWAS direction	Direction concordance	Novel variant
		REF/REF	REF/ALT	ALT/ALT	REF/REF	REF/ALT	ALT/ALT					
*COL4A4*	2:227089883:G:A	19 219	36 985	18 013	9638	18 917	9532	8.99E-03	Myopia	Myopia	−−	Yes
*RASGEF1B*	4:81448051:T:A	29 046	34 529	10 633	14 984	17 921	5177	3.32E-03	Hyperopia	Hyperopia	++	Yes
*ARMS2*	10:122454932:G:T	46 081	24 637	3493	23 436	12 889	1752	8.19E-02	Myopia	Myopia	−−	Yes
*ATM*	11:108258930:A:G	22 705	35 749	15 047	11 577	18 324	7840	4.69E-01	Myopia	Myopia	−−	Yes
*BMP4*	14:53950804:A:G	24 538	36 256	13 449	12 335	18 639	7125	1.61E-02	Myopia	Myopia	−−	Yes
*BMP4*	14:53951768:C:T	74 231	11	−	38 095	2	−	x	−	Myopia	x	Yes
*SIX6*	14:60509783:G:A	73 247	996	−	37 481	615	−	3.10E-04	Myopia	Myopia	−−	Yes
*SIX6*	14:60509819:C:A	27 857	35 412	10 974	13 990	18 205	5905	1.49E-03	Myopia	Myopia	−−	Yes
*GSDMA*	17:39966427:G:T	18 936	37 034	18 260	9602	19 103	9392	5.28E-01	Myopia	Myopia	−−	Yes
*GNGT2*	17:49207373:T:C	66 044	7953	234	33 729	4218	147	2.71E-02	Myopia	Myopia	−−	Yes
*ZNF652*	17:49312652:G:C	34 966	32 049	7120	17 699	16 557	3791	4.82E-02	Myopia	Myopia	−−	Yes
*CRX*	19:47836338:G:A	73 619	624	−	37 860	240	−	1.12E-04	Hyperopia	Hyperopia	++	Yes
*PDE11A*	2:177701185:T:C	68 473	5631	−	34 760	3228	−	1.51E-07	Myopia	Myopia	−−	No
*PRSS56*	2:232520686:G:A	36 488	31 188	6539	17 976	16 284	3827	7.61E-15	Myopia	Myopia	−−	No
*PRSS56*	2:232523470:G:T	73 937	306	−	37 922	178	−	1.94E-01	Myopia	Hyperopia	−+	No
*BMP3*	4:81031483:T:A	70 089	4093	−	35 682	2377	−	8.44E-07	Myopia	Myopia	−−	No
*RGR*	10:84252957:C:T	30 958	33 924	9357	15 283	17 696	5121	1.02E-07	Myopia	Myopia	−−	No
*KAZALD1*	10:101064592:G:C	32 571	33 134	8538	17 001	16 960	4139	2.10E-03	Hyperopia	Hyperopia	++	No
*GNB3*	12:6845700:G:A	64 401	9494	347	32 730	5164	205	3.47E-04	Myopia	Myopia	−−	No

x = Too few counts for reliable validation test calculation. Abbreviations: Variant = gnomAD format identifier (Chromosome:Position:REF:ALT); REF = reference allele present in GRCh38; ALT = alternate allele (this is the minor allele for the variants listed here). REF/REF = participants carrying zero copies of the ALT allele; REF/ALT = participants carrying one copy of the ALT allele; ALT/ALT = participants carrying two copies of the ALT allele; *P* = *P*-value for Chi-squared or Fisher’s test; Direction concordance = direction of effect in GWAS and validation analyses.

### Contribution of rare exonic variants to refractive error

Statistical power to detect variants associated with refractive error in the current sample varied as a function of the risk allele’s effect size and frequency (Supplementary Material, Fig. S12). Power was approximately 100% to detect variants with an effect size of −5.00 D, which would be sufficient to cause monogenic high myopia, only if the variant was present in >40 individuals in the GWAS sample (MAF ≈ 0.0004). When variants were present in at least 400 different individuals (MAF ≈ 0.004), power was approximately 25 and 100% to detect variants with effect sizes of −0.50 D and − 1.00 D, respectively. Accordingly, the current study suggested that no exonic variants with effect sizes >±1.00 D and MAF > 0.004 exist in Europeans. In other words, the current work suggests commonly occurring variants with large effects on refractive error do not exist, at least within coding regions of the human genome of Europeans.

## Discussion

Our WES GWAS analysis and fine-mapping study implicated two novel genes as conferring susceptibility to refractive error (*COL4A4* and *ATM*). However, we caution that of these two genes, only the lead variant in *COL4A4* demonstrated evidence of association in the *AOSW* case–control validation sample (Table 4), leaving open the possibility that the association with *ATM* was a false-positive finding. More convincingly, the current study led to 19 specific genetic variants being prioritized as putative causal variants, 12 of which were novel discoveries. Of the 12 novel putative causal variants, 11 could be tested for independent validation and 8 (73%) of these variants displayed at least nominal evidence of association (*P* < 0.05) with *AOSW* case–control status in the validation sample (Table 4). Furthermore, the variants associated with a more myopic refractive error in the GWAS were associated with a greater likelihood of having spectacles between 6 and 25 years of age, while variants associated with a more hyperopic refractive error in the GWAS were associated with a lower likelihood of having spectacles during this age range (Table 4 and Supplementary Material, Table S4). This high level of concordance for the direction of effect (100% concordance for 11 novel putative causal variants; 95% concordance for all 18 putative causal variants that could be tested) suggested the bulk of the associations reported in the current study were robust findings.

In past GWAS studies, the majority of genome-wide significant variants have been found in non-coding regions of the genome and thus it has not been straightforward to assign lead GWAS variants to specific genes. In contrast, by focusing on pLOF and missense variants in exons, the current WES GWAS study more clearly implicated specific genes as putative myopia susceptibility genes. The discovery of two independently associated variants in the *PRSS56*, *BMP4* and *SIX6* genes provided additional evidence that these genes play a role in the development of refractive errors. By performing conditional analyses, in which we controlled for the effects of the lead fine-mapped variant in each region, we were able to infer whether the putative causal variant fully explained the GWAS signal in that region (17). Another important insight gained in the present study by examining rare variants was that it was often possible to infer whether a risk allele conferred susceptibility to myopia or to hyperopia (Fig. 1; Table 3). Past GWAS analyses of commonly occurring variants have generally been unable to address this question, except in the unusual event that a common variant has a dominant or recessive mode of action (18,19).

Simulations suggested our WES GWAS had insufficient statistical power to identify rare variants with effect sizes large enough to cause monogenic high myopia unless these alleles were present in at least 1 per 1250 individuals (MAF > 0.0004). This underscores the strengths and weaknesses of community-based resources, such as UK Biobank, for studying the genetic architecture of diseases. A population-based sample provides the opportunity to determine the frequencies of myopia risk-conferring alleles in the population, yet ultimately a high myopia case–control WES study, including many thousands of cases and controls (or a pedigree-based WES study), is required to identify specific rare variants responsible for causing monogenic high myopia.

This is the first large-scale search for pLOF and missense exonic variants associated with refractive error. It was noteworthy that most of the regions identified as being associated with refractive error were those already reported in past GWAS analyses for refractive error rather than in genes previously identified in sequencing studies of pedigrees and probands with high myopia (20–23). This emphasizes the limited role any particular rare variant plays in contributing to high myopia in the general population. High-quality genotyping of both common and rare exonic variants facilitated fine-mapping, which resulted in the novel discovery of 12 putative causal variants in 10 genes as well as 2 novel candidate genes associated with refractive error. A rare missense variant in *CRX* (19:47836338:G:A) previously thought to be a benign was associated with a +0.70 D more hyperopic refractive error, on average, and, independently, associated with a lower likelihood of first wearing spectacles at 6–25 years of age. The pathway through which this *CRX* variant impacts refractive error may therefore hold promise as a therapeutic target for inhibiting the progression of myopia.

## Materials and Methods

Details of the analysis methods are provided in the Supplement.

### GWAS sample and AOSW case–control validation sample

The GWAS analysis sample and the validation analysis sample were drawn from participants of UK Biobank, a longitudinal study of health and well-being that enrolled 500 000 UK citizens aged 40–69 years between 2006 and 2010 (24). The North-West Research Ethics Committee approved the study (reference, 06/MRE08/65) in accordance with the principles of the Declaration of Helsinki. All participants gave written informed consent. UK Biobank participants provided a blood sample, which was processed for array-based genotyping of ~ 800 000 genetic markers (25). Additional genotypes were imputed using a merged HRC reference panel and a combined UK10K and 1000 Genomes phase 3 reference panel, as described (25). These genotype data along with familial relatedness information and ancestry principal component (PC) loadings were released by the UK Biobank team to authorized researchers in July 2016 (genomic coordinates specified according to build GRCh37). The quality of the genotype imputation for the UK Biobank samples has been reported to be high: INFO ≈ 0.8 for variants with MAF 0.01 and INFO > 0.95 for variants with MAF > 0.05 (25). WES of samples and quality control processing were performed by the UK Biobank team as described (26,27). WES data for 200 000 UK Biobank participants were released by the UK Biobank team in October 2020 (genomic coordinates specified according to build GRCh38). WES coverage in the UK Biobank has been reported to exceed 20× at 94.6% of sites on average (26), with 97.2% of the consensus coding sequence covered with at least 10× coverage (27). This coverage was sufficient to provide high-quality genotype calls (26,27). Ocular measurements, which included non-cycloplegic autorefraction, were introduced in the latter stages of UK Biobank recruitment such that only 23% of participants were assessed (28). However, all participants were asked their AOSW. The GWAS analysis sample was restricted to participants with data available for avMSE and WES. Following Pozarickij *et al*. (8), further exclusions were applied to limit the sample to unrelated individuals of European genetic ancestry, with no self-reported history or hospital-record history of an eye disorder that could influence refractive error. This resulted in a sample of *n* = 51 624 unrelated individuals. An independent group of *n* = 112 343 unrelated WES-genotyped UK Biobank participants of European genetic ancestry with information on AOSW but without avMSE information were used as a validation sample. Based on the reported (18) relationship between avMSE and AOSW, individuals in the validation sample were classified as cases if they had an AOSW > 5 years and ≤25 years and as controls otherwise.

### GWAS for refractive error

pLOF variants were defined according to the criteria of Van Hout (26): namely, ‘stop gained’, ‘stop lost’, ‘start lost’, ‘splice donor’, ‘splice acceptor’ and ‘frameshift’. Linear regression analyses with the RINT-transformed avMSE phenotype were performed using PLINK v1.9 (29). Genotypes were coded as 0, 1 or 2 according to counts of minor alleles. Age, age-squared, sex and the first 10 ancestry PCs were included as covariates. Following Van Hout (26), only genetic variants with a minor allele count (MAC) of 4 or above and a genotype missing rate <0.02 were included in the analysis. A total of 524 442 genetic variants met these criteria, including 29 179 pLOF variants and 495 263 missense variants. Rare variants were defined as those with MAF < 0.01. QQ plots for a trait simulated under the null hypothesis of no association with the genetic variants demonstrated good agreement between the expected and observed *P*-value distribution (Supplementary Material, Fig. S2). To account for the large number of statistical tests carried out, a correction for multiple testing was undertaken by applying the Bonferroni method. Specifically, an association was considered significant if *P* < 9.5E-08 (0.05/524442 = 9.53E-08).

### Fine-mapping analysis, conditional analyses and validation analysis

The R package SUSIE (14) was used to fine-map regions ±500 kb either side of the lead variants identified in the GWAS. The RINT-transformed avMSE phenotype was regressed on the same set of covariates used in the GWAS analysis, and the residuals were tested for association using *SUSIE*. All WES variants with a MAC of at least 4 and a missing genotype rate <0.02 were included. Conditional GWAS analyses were performed for array-genotyped and imputed variants with a MAC of at least 4 and a missing genotype rate <0.02 with PLINK v2.0 (29). Regions ±500 kb either side of the lead variants were analyzed before and after conditioning on the lead WES variant in the region. To aid interpretation, the effect sizes of variants associated with refractive error are reported in units of D per copy of the effect allele in the main text and in Table 3 rather than in units of standard deviations of RINT-transformed avMSE per copy of the effect allele. Effect sizes in D per copy of the effect allele were calculated by re-running the GWAS regression analysis for individual variants using untransformed avMSE as the dependent variable.

To independently validate the association of the lead variants with refractive error, either a 3 × 2 or a 2 × 2 table was constructed of the genotype counts in AOSW cases and controls (note, for very rare variants, there were no individuals homozygous for the minor allele, thus yielding a 2 × 2 table rather than a 3 × 2 table). A difference in genotype counts between cases and controls was performed using Fisher’s exact test for 2 × 2 tables and a chi-squared test for 3 × 2 tables. No covariates were included in these validation tests.

### Power calculation

Simulations were used to assess statistical power to detect variants associated with refractive error as a function of the risk allele effect size and MAC. Details are provided in the Supplement.

## Supplementary Material

HMG_R2_supplement_ddac004
