# Supplementary material for: Whole exome sequence analysis in 51 624 participants identifies novel genes and variants associated with refractive error and myopia
Source: Hum Mol Genet. 2022 Jan 12;31(11):1909–19. doi: 10.1093/hmg/ddac004 (PMC9169456; doi:10.1093/hmg/ddac004)
Supplement: HMG_R2_supplement_ddac004 [file hmg_r2_supplement_ddac004.pdf]

Whole exome sequence analysis in 51,624 participants identifies novel genes and variants associated with refractive error and myopia

Online Supplemental Materials

Item	Title	Page
eMethods	Online supplementary methods	2
Table S1	Lead pLOF and missense WES variants identified in GWAS for refractive error.	5
Table S2	Fine mapping of GWAS regions identified using WES data.	6
Table S3	Demographic characteristics of the validation sample.	7
Table S4	Summary of fine mapping and validation analysis results.	8
Figure S1	Quantile-quantile plots of the GWAS for refractive error.	9
Figure S2	Quantile-quantile plots of the GWAS for a trait simulated under the null hypothesis of no association with the genetic variants	10
Figure S3	Distribution of refractive error in individuals carrying pLOF or missense variants in *SIX6, CRX, C4orf47, BMP4, GOLGA8H, TRPM1, PDE11A* and *GNGT2*.	11
Figure S4	Fine-mapping and conditional analysis: *BMP4*.	12
Figure S5	Fine-mapping and conditional analysis: *SIX6* and *CRX*.	13
Figure S6	Fine-mapping and conditional analysis: *COL4A4* and *BMP3-RASGEF1B*.	14
Figure S7	Fine-mapping and conditional analysis: *ARMS2* and *NPAT-ATM.*	15
Figure S8	Fine-mapping and conditional analysis: *GSDMA* and *GNGT2- ZNF652.*	16
Figure S9	Fine-mapping and conditional analysis: *PDE11A* and *RGR.*	17
Figure S10	Fine-mapping and conditional analysis: *PRSS56* and *KAZALD1.*	18
Figure S11	Fine-mapping and conditional analysis: *GNB3.*	19
Figure S12	Power to detect a genetic variant associated with refractive error as a function of effect size and minor allele count (MAC).	20

Online Supplementary Methods

GWAS analysis sample and validation sample

The spherical equivalent refractive error was averaged between the right and left eyes of each participant ('*avMSE*'). The GWAS analysis sample was restricted to participants with data available for *avMSE* and WES. Only participants of European ancestry were studied, since the available sample size for other ancestry groups was under-powered for a GWAS analysis. Following Pozarickij et al. (1), further exclusions were applied to limit the sample to individuals with genetic heterozygosity within 4 standard deviations of the European cohort mean, matching genetically-inferred and self-reported sex, and no self-reported history or hospital-record history of an eye disorder that could influence refractive error. Participants were also excluded if they were recruited from an assessment center that provided ophthalmic data for fewer than 50 individuals. After the above exclusions, the maximum set of unrelated participants was selected using the *R* package *igraph* (https://igraph.org/r/). This resulted in a sample of n=51,624 unrelated individuals.

The validation sample was selected from amongst participants of European genetic ancestry, genetic heterozygosity within 4 standard deviations of the European cohort mean, and matching genetically-inferred and self-reported sex, who had either self-reported their age-of-onset of spectacle wear (*AOSW*) or self-reported that they did not wear spectacles. Cases were defined as individuals with an *AOSW* > 5 years and ≤ 25 years. All other individuals, including those who reported not wearing spectacles, were classified as controls. Individuals were excluded if they were related to any of the 51,624 participants in the GWAS sample. After the above exclusions, the maximum set of unrelated participants was selected, resulting in a validation sample of n=112,343 participants (38,100 cases and 74,243 controls).

GWAS analysis for avMSE

Genetic variants were annotated with SnpEff (2), using the xgen_plus_spikein.b38.chr[1-22].bed and GRCh38.86 reference databases. Putative loss-of-function (pLOF) variants were defined according to the criteria of Van Hout (3): namely, 'stop gained', 'start lost', 'splice donor', 'splice acceptor' and 'frameshift'. Tests for association between the *avMSE* refractive error phenotype and the genotype of genetic variants in the sample of unrelated participants were performed separately for each marker using *PLINK* v1.9 (4). Simulations demonstrated that GWAS analysis of the untransformed *avMSE* phenotype led to an excess of false positives results; specifically, an excess of spurious, very highly significant associations with rare variants. To avoid these false positives, it was necessary to rank-inverse-normal transform (RINT) *avMSE* prior to analysis.

Genotypes were coded 0, 1 or 2, corresponding to the count of minor alleles carried by an individual. Age, age^2 and the first 10 PCs and sex (coded as a binary variable) were included as covariates. The age, age^2 and PC variables were all standardized to have a mean of zero and a standard deviation of one, in order to reduce collinearity and facilitate model fitting. Only genetic variants with a minor allele count (MAC) of 4 or above in the sample and a genotype missing rate < 0.02 were included in the analysis, which yielded a total of 29,179 pLOF variants and 495,263 missense variants. MAF values reported in the text and tables were calculated in the GWAS analysis sample. Variants were taken forward for fine-mapping if they had a p-value <0.05/m, where m was the number of variants tested (m = 29,179 + 495,263 = 524,442).

Fine mapping

Fine mapping was performed with *SUSIE* (5) in the sample of n=51,624 unrelated individuals used for the single marker GWAS analysis. All WES variants within a region ±500 kb from the lead variants that had a minor allele count (MAC) of at least 4 and a genotype missing rate < 0.02 were included in the fine-mapping analysis. The residuals from the RINT-transformed *avMSE* phenotype, regressed on age, age^2, sex and the first 10 PCs, was used as the dependent variable. Genotypes were coded as the counts of minor alleles (0, 1 or 2). The analyses allowed for up to 10 independent putative causal variants in each region. Graphs of the fine-mapping results were created and aligned in *R* using the packages *ggplot2*, *cowplot*, and *egg*, and gene co-ordinates downloaded from Ensembl biomart (https://www.ensembl.org/biomart/) for genome build GRCh38.p13.

Conditional analyses

Regional GWAS analyses were performed using *PLINK*, for regions spanning ±500 kb from the lead variants. The same GWAS parameters and covariates were adopted as in the original GWAS except that the genotype of the lead variant was included as an additional covariate. The *vif* and *max-corr* settings of *PLINK* were adjusted to allow GWAS variants to be tested even if they were in strong LD with the lead conditioning variant. The UCSC *Liftover* tool was used to convert between GRCh37 genomic coordinates (imputed genotypes) and GRCh38 genomic coordinates (WES genotypes).

Statistical power calculations

Simulations were performed to quantify the statistical power of the WES GWAS analysis. A set of 4, 40, 100 or 400 participants from the GWAS sample of unrelated participants (n = 51,624) were randomly assigned as heterozygous for a putative risk variant; unselected participants were assigned as homozygous for the non-risk allele. The true refractive error of heterozygotes

was modified by a fixed value (0.00, -0.50, -1.00 or -5.00 D) mimicking the effect of the risk allele. The trait was RINT-transformed, and a linear regression test for association between RINT-transformed *avMSE* and the simulated genotype was performed, with the same set of covariates used in the true GWAS analysis. The simulation was repeated 1000 times for each parameter setting. The proportion of tests with $P < 9.53E-08$ for association with the risk variant was evaluated as a function of MAC and effect size.

Table S1. Lead pLOF and missense WES variants identified in GWAS for refractive error. Shading indicates variants located in the same region.

Gene	Category	Variant	HGVS	rsID	Chr	Pos	REF	ALT	EA	MAF	Beta	SE	P	Anno
SIX6	Rare	14:60509783:G:A	ENST00000327720.5:c.385G>A (p.Glu129Lys)	rs146737847	14	60509783	G	A	A	0.007	-0.302	0.037	1.68E-16	missense
CRX	Rare	19:47836338:G:A	ENST00000221996.11:c.196G>A (p.Val66Ile)	rs61748438	19	47836338	G	A	A	0.004	0.288	0.049	4.92E-09	missense
PDE11A	Common	2:177701185:T:C	ENST00000286063.10:c.2180A>G (p.Tyr727Cys)	rs17400325	2	177701185	T	C	C	0.041	-0.107	0.015	2.31E-12	missense
COL4A4	Common	2:227089883:G:A	ENST00000396625.3:c.1444C>T (p.Pro482Ser)	rs2229814	2	227089883	G	A	A	0.499	-0.035	0.006	1.06E-08	missense
ALPP	Common	2:232378876:C:T	ENST00000392027.2:c.74C>T (p.Pro25Leu)	rs1130335	2	232378876	C	T	T	0.081	0.070	0.011	2.23E-10	missense
ALPG	Common	2:232409765:C:A	ENST00000295453.7:c.1492C>A (p.Arg498Ser)	rs56080708	2	232409765	C	A	A	0.084	0.072	0.011	2.97E-11	missense
PRSS56	Common	2:232520686:G:A	ENST00000449534.6:c.88G>A (p.Ala30Thr)	rs1550094	2	232520686	G	A	G	0.304	-0.068	0.007	1.09E-24	missense
BMP3	Common	4:81031483:T:A	ENST00000282701.2:c.199T>A (p.Tyr67Asn)	rs74764079	4	81031483	T	A	A	0.028	-0.117	0.018	2.08E-10	missense
LRIT2	Common	10:84222045:T:G	ENST00000538192.4:c.1558A>C (p.Thr520Pro)	rs6585847	10	84222045	T	G	G	0.475	0.033	0.006	7.13E-08	missense
KAZALD1	Common	10:101064592:G:C	ENST00000370200.5:c.764G>C (p.Gly255Ala)	rs807037	10	101064592	G	C	G	0.336	0.039	0.006	1.36E-09	missense
ARMS2	Common	10:122454932:G:T	ENST00000528446.1:c.205G>T (p.Ala69Ser)	rs10490924	10	122454932	G	T	T	0.216	-0.040	0.007	4.39E-08	missense
NPAT	Common	11:108173261:C:T	ENST00000278612.8:c.1723G>A (p.Val575Ile)	rs2070661	11	108173261	C	T	C	0.436	-0.033	0.006	5.53E-08	missense
GNB3	Common	12:6845700:G:A	ENST00000435982.6:c.811G>A (p.Gly271Ser)	rs5442	12	6845700	G	A	A	0.070	-0.097	0.012	3.57E-16	missense
BMP4	Common	14:53950804:A:G	ENST00000558961.1:c.313T>C (p.Ter105ArgextTer26)	rs17563	14	53950804	A	G	A	0.424	-0.037	0.006	2.25E-09	pLOF
C14orf39	Common	14:60437039:G:A	ENST00000321731.7:c.1570C>T (p.Leu524Phe)	rs1254319	14	60437039	G	A	A	0.291	-0.039	0.007	4.83E-09	missense
SIX6	Common	14:60509819:C:A	ENST00000327720.5:c.421C>A (p.His141Asn)	rs33912345	14	60509819	C	A	C	0.387	-0.041	0.006	5.03E-11	missense
GSDMA	Common	17:39965740:G:A	ENST00000301659.8:c.53G>A (p.Arg18Gln)	rs3894194	17	39965740	G	A	A	0.455	-0.036	0.006	2.44E-09	missense
GSDMA	Common	17:39966427:G:T	ENST00000301659.8:c.382G>T (p.Val128Leu)	rs7212938	17	39966427	G	T	G	0.499	-0.038	0.006	3.97E-10	missense
GNGT2	Common	17:49207373:T:C	ENST00000300406.6:c.50A>G (p.Gln17Arg)	rs35638197	17	49207373	T	C	C	0.059	-0.070	0.013	3.91E-08	missense

Abbreviations: pLOF=putative loss of function; MAF=Minor allele frequency; HGVS= Human Genome Variation Society nomenclature format; Chr=Chromosome; Pos=Genomic position in GRCh38 coordinates; EA=effect allele; Beta=Regression coefficient for RINT-*avMSE* per copy of the effect allele; rsID=dbSNP reference ID

Table S2. Fine-mapping of GWAS regions identified using WES data. Shading indicates independently-associated variants located in the same region.

Gene	Variant	HGVS	rsID	Chr	Pos	PIP	P	MAF	Anno	Novel gene?	Novel variant?
PDE11A	2:177701185:T:C	ENST00000286063.10:c.2180A>G (p.Tyr727Cys)	rs17400325	2	177701185	1.000	4.00E-12	0.041	missense	(6)	No
COL4A4	2:227089883:G:A	ENST00000396625.3:c.1444C>T (p.Pro482Ser)	rs2229814	2	227089883	0.960	1.03E-08	0.499	missense	Yes	Yes
PRSS56	2:232520686:G:A	ENST00000449534.6:c.88G>A (p.Ala30Thr)	rs1550094	2	232520686	1.000	1.21E-24	0.304	missense	(6)	No
PRSS56	2:232523470:G:T	ENST00000449534.6:c.904G>T (p.Val302Phe)	rs74703359	2	232523470	0.996	1.09E-07	0.002	missense	(7)	No
BMP3	4:81031483:T:A	ENST00000282701.2:c.199T>A (p.Tyr67Asn)	rs74764079	4	81031483	1.000	1.64E-10	0.028	missense	(6)	No
RASGEF1B	4:81448051:T:A	ENST00000264400.6:c.654+18A>T (intronic)	rs1077803	4	81448051	0.692	3.74E-08	0.356	intronic	(8)	Yes
RGR	10:84252957:C:T	ENST00000359452.8:c.471C>T (p.Tyr157Tyr)	rs1042454	10	84252957	0.783	2.28E-12	0.363	synonymous	(6)	No
KAZALD1	10:101064592:G:C	ENST00000370200.5:c.764G>C (p.Gly255Ala)	rs807037	10	101064592	1.000	1.35E-09	0.336	missense	(9)	No
ARMS2	10:122454932:G:T	ENST00000528446.1:c.205G>T (p.Ala69Ser)	rs10490924	10	122454932	0.791	2.79E-08	0.216	missense	(10)	Yes
ATM	11:108258930:A:G	ENST00000675843.1:c.2377-56A>G (intronic)	rs672655	11	108258930	0.439	1.19E-08	0.433	intronic	Yes	Yes
GNB3	12:6845700:G:A	ENST00000435982.6:c.811G>A (p.Gly271Ser)	rs5442	12	6845700	1.000	3.49E-16	0.07	missense	(9)	No
BMP4	14:53950804:A:G	ENST00000558961.1:c.313T>C (p.Ter105ArgextTer26)	rs17563	14	53950804	1.000	2.39E-09	0.424	pLOF	(6)	Yes
BMP4	14:53951768:C:T	ENST00000245451.9:c.370+85G>A (intronic)	rs534912805	14	53951768	0.530	1.89E-04	0.0002	intronic	(6)	Yes
SIX6	14:60509783:G:A	ENST00000327720.5:c.385G>A (p.Glu129Lys)	rs146737847	14	60509783	1.000	1.65E-16	0.007	missense	(10)	Yes
SIX6	14:60509819:C:A	ENST00000327720.5:c.421C>A (p.His141Asn)	rs33912345	14	60509819	0.798	5.29E-11	0.387	missense	(10)	Yes
GSDMA	17:39966427:G:T	ENST00000301659.8:c.382G>T (p.Val128Leu)	rs7212938	17	39966427	0.568	4.95E-10	0.499	missense	(10)	Yes
GNGT2	17:49207373:T:C	ENST00000300406.6:c.50A>G (p.Gln17Arg)	rs35638197	17	49207373	0.981	6.05E-08	0.059	missense	(10)	Yes
ZNF652	17:49312652:G:C	ENST00000430262.3:c.1048+46C>G (intronic)	rs2072153	17	49312652	0.785	4.58E-07	0.300	intronic	(10)	Yes
CRX	19:47836338:G:A	ENST00000221996.11:c.196G>A (p.Val66Ile)	rs61748438	19	47836338	0.999	4.89E-09	0.004	missense	(10)	Yes

Abbreviations: rsID=dbSNP reference ID; HGVS= Human Genome Variation Society nomenclature format; PIP=Posterior inclusion probability; P=P-value from *SUSIE* regression analysis.

Table S3. Demographic characteristics of the validation sample. Unless stated otherwise, values are median (25th percentile to 75th percentile). P-value is for a chi-squared test examining the proportion of the sample who were female or a Wilcoxon test examining the other traits in cases versus controls.

**Variable**		**All**	**Controls**	**Cases**	***P***
N		112,343	74,243	38,100	-
Female (%)		55.1%	53.1%	58.9%	9.70E-76
Age (years)	Median (IQR)	58.42 (50.67 to 63.83)	58.50 (50.50 to 63.92)	58.33 (50.83 to 63.58)	3.40E-02
Age-of-onset Spectacle Wear (years)	Median (IQR)	35.00 (15.00 to 46.00)	45.00 (40.00 to 50.00)	14.00 (10.00 to 18.00)	<1.0E-99
Age completed education (years)	Median (IQR)	17.00 (16.00 to 21.00)	17.00 (16.00 to 21.00)	18.00 (16.00 to 21.00)	<1.0E-99
Height (m)	Median (IQR)	1.68 (1.62 to 1.75)	1.68 (1.62 to 1.75)	1.68 (1.61 to 1.75)	6.30E-18

Table S4. Summary of fine mapping and validation analysis results.

Gene	Variant	Discovery GWAS for refractive error				SUSIE fine mapping		Validation case-control analysis for association with early AOSW										Direction concordance	Novel variant
		Beta	SE	*P*	GWAS direction	PIP	*P*	Controls REF/REF	Controls REF/ALT	Controls ALT/ALT	Cases REF/REF	Cases REF/ALT	Cases ALT/ALT	*P*	MAF in cases	MAF in controls	Validation direction		
COL4A4	2:227089883:G:A	-0.035	0.006	1.06E-08	Myopia	0.960	1.03E-08	19219	36985	18013	9638	18917	9532	8.99E-03	0.499	0.492	Myopia	--	Yes
RASGEF1B	4:81448051:T:A	0.035	0.006	2.89E-08	Hyperopia	0.692	3.74E-08	29046	34529	10633	14984	17921	5177	3.32E-03	0.371	0.376	Hyperopia	++	Yes
ARMS2	10:122454932:G:T	-0.040	0.007	4.39E-08	Myopia	0.791	2.79E-08	46081	24637	3493	23436	12889	1752	8.19E-02	0.215	0.213	Myopia	--	Yes
ATM	11:108258930:A:G	-0.035	0.006	1.20E-08	Myopia	0.439	1.19E-08	22705	35749	15047	11577	18324	7840	4.69E-01	0.450	0.448	Myopia	--	Yes
BMP4	14:53950804:A:G	-0.037	0.006	2.25E-09	Myopia	1.000	2.39E-09	24538	36256	13449	12335	18639	7125	1.61E-02	0.432	0.425	Myopia	--	Yes
BMP4	14:53951768:C:T	-0.414	0.308	1.79E-01	Myopia	0.530	1.89E-04	74231	11	-	38095	2	-	x	2.62E-05	7.41E-05	Hyperopia	x	Yes
SIX6	14:60509783:G:A	-0.302	0.037	1.68E-16	Myopia	1.000	1.65E-16	73247	996	-	37481	615	-	3.10E-04	0.00807	0.00671	Myopia	--	Yes
SIX6	14:60509819:C:A	-0.041	0.006	5.03E-11	Myopia	0.798	5.29E-11	27857	35412	10974	13990	18205	5905	1.49E-03	0.394	0.386	Myopia	--	Yes
GSDMA	17:39966427:G:T	-0.038	0.006	3.97E-10	Myopia	0.568	4.95E-10	18936	37034	18260	9602	19103	9392	5.28E-01	0.497	0.495	Myopia	--	Yes
GNGT2	17:49207373:T:C	-0.070	0.013	3.91E-08	Myopia	0.981	6.05E-08	66044	7953	234	33729	4218	147	2.71E-02	0.0592	0.0567	Myopia	--	Yes
ZNF652	17:49312652:G:C	-0.034	0.007	2.82E-07	Myopia	0.785	4.58E-07	34966	32049	7120	17699	16557	3791	4.82E-02	0.317	0.312	Myopia	--	Yes
CRX	19:47836338:G:A	0.288	0.049	4.92E-09	Hyperopia	0.999	4.89E-09	73619	624	-	37860	240	-	1.12E-04	0.00315	0.00420	Hyperopia	++	Yes
PDE11A	2:177701185:T:C	-0.107	0.015	2.31E-12	Myopia	1.000	4.00E-12	68473	5631	-	34760	3228	-	1.51E-07	0.0425	0.038	Myopia	--	No
PRSS56	2:232520686:G:A	-0.068	0.007	1.09E-24	Myopia	1.000	1.21E-24	36488	31188	6539	17976	16284	3827	7.61E-15	0.314	0.298	Myopia	--	No
PRSS56	2:232523470:G:T	0.338	0.064	1.10E-07	Hyperopia	0.996	1.09E-07	73937	306	-	37922	178	-	1.94E-01	0.00234	0.00206	Myopia	-+	No
BMP3	4:81031483:T:A	-0.117	0.018	2.08E-10	Myopia	1.000	1.64E-10	70089	4093	-	35682	2377	-	8.44E-07	0.0312	0.0276	Myopia	--	No
RGR	10:84252957:C:T	-0.044	0.006	2.57E-12	Myopia	0.783	2.28E-12	30958	33924	9357	15283	17696	5121	1.02E-07	0.367	0.355	Myopia	--	No
KAZALD1	10:101064592:G:C	0.039	0.006	1.36E-09	Hyperopia	1.000	1.35E-09	32571	33134	8538	17001	16960	4139	2.10E-03	0.331	0.338	Hyperopia	++	No
GNB3	12:6845700:G:A	-0.097	0.012	3.57E-16	Myopia	1.000	3.49E-16	64401	9494	347	32730	5164	205	3.47E-04	0.0732	0.0686	Myopia	--	No

x – Too few counts for reliable Fisher's test calculation.

Abbreviations. Variant=gnomAD format identifier (Chromosome:Position:REF:ALT); Beta=Beta coefficient in units of standard deviations of RINT-transformed *avMSE* per copy of the ALT allele; SE=Standard error of beta coefficient; P=P-value; GWAS direction=Direction of risk associated with ALT allele; PIP=Posterior inclusion probability; REF=Reference allele present in GRCh38; ALT=Alternate allele (this is the minor allele for the 36 variants listed here). REF/REF=Participants carrying 0 copies of the ALT allele; REF/ALT=Participants carrying 1 copy of the ALT allele; ALT/ALT=Participants carrying 2 copies of the ALT allele; MAF=Frequency of minor (ALT) allele; Direction concordance=Direction of effect in GWAS and validation analyses.

Figure S1. Quantile-quantile plots of the GWAS for refractive error. pLOF and missense variants were stratified by MAF to illustrate the lack of an excess of association for ultra-rare variants. The red line indicates the expected distribution of p-values under the null hypothesis (associations occurring due to chance rather than due to association with refractive error).

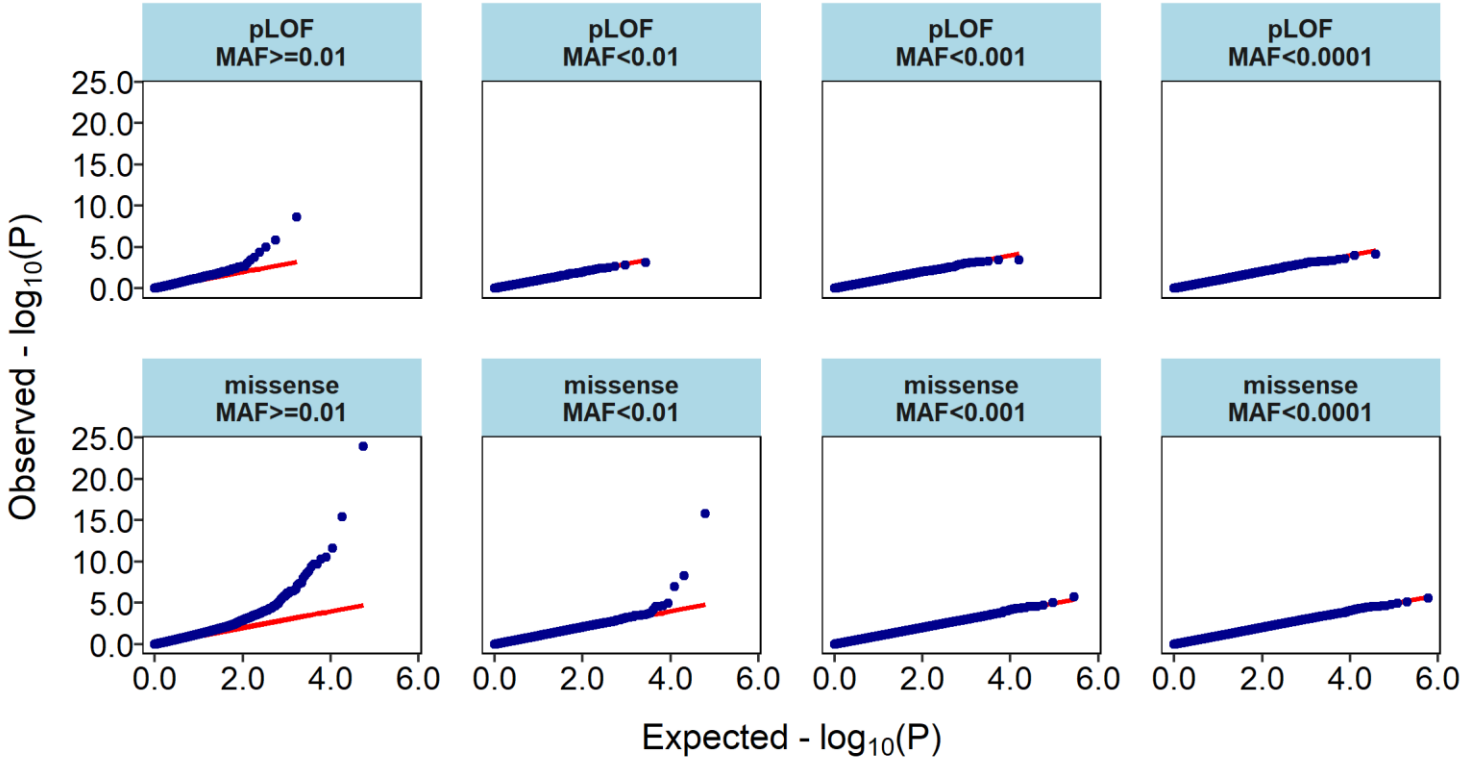

Figure S2. Quantile-quantile plots of the GWAS for a trait simulated under the null hypothesis of no association with the genetic variants. pLOF and missense variants were stratified by MAF. The red line indicates the expected distribution of p-values under the null hypothesis.

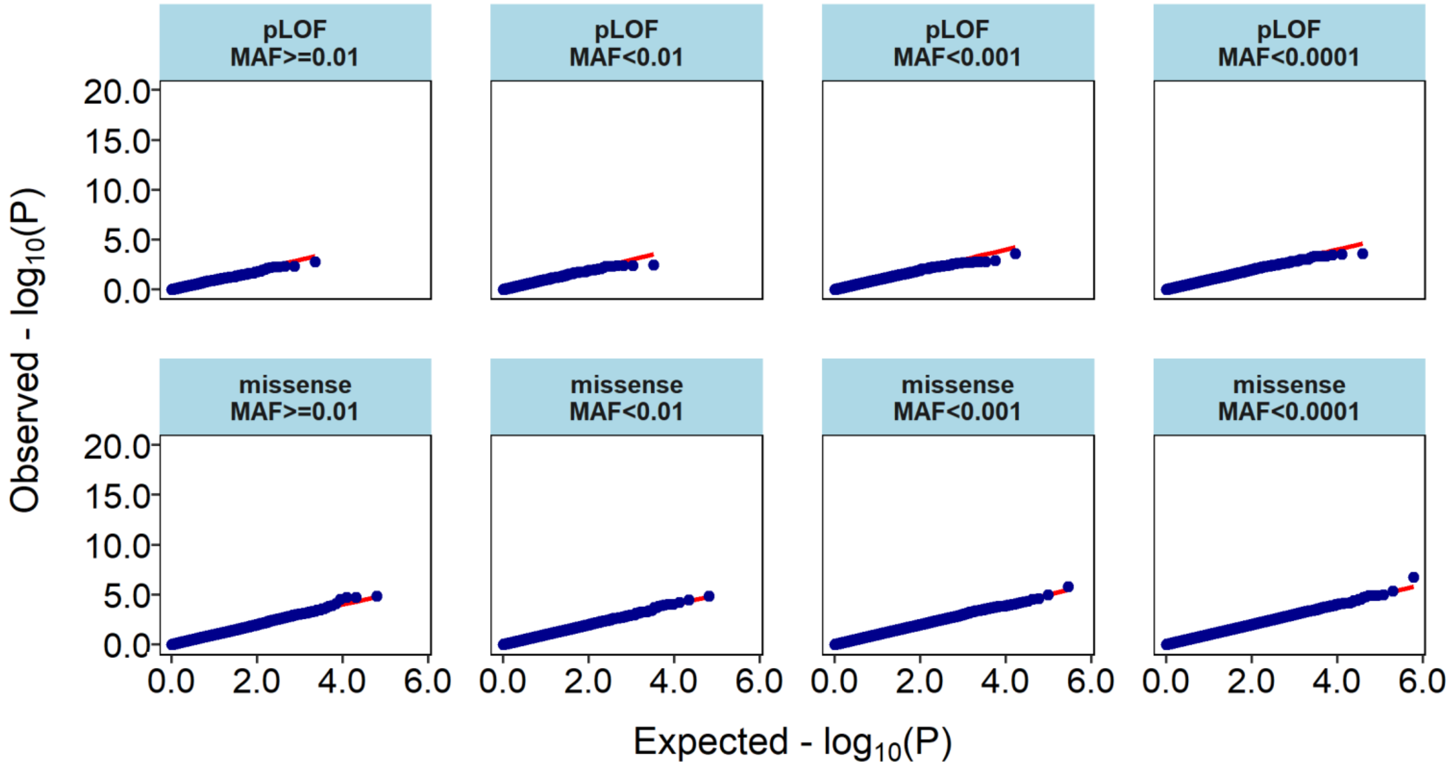

Figure S3. Distribution of refractive error in individuals carrying pLOF or missense variants in *PDE11A, BMP3, BMP4, SIX6, GNGT2* and *CRX*. Note that certain risk alleles are associated with a more myopic refractive error, such as the 'A' allele of *SIX6* variant 14:60509783:G:A, while other risk alleles are associated with a more hyperopic refractive error, such as the 'A' allele of *CRX* variant 19:47836338:G:A.

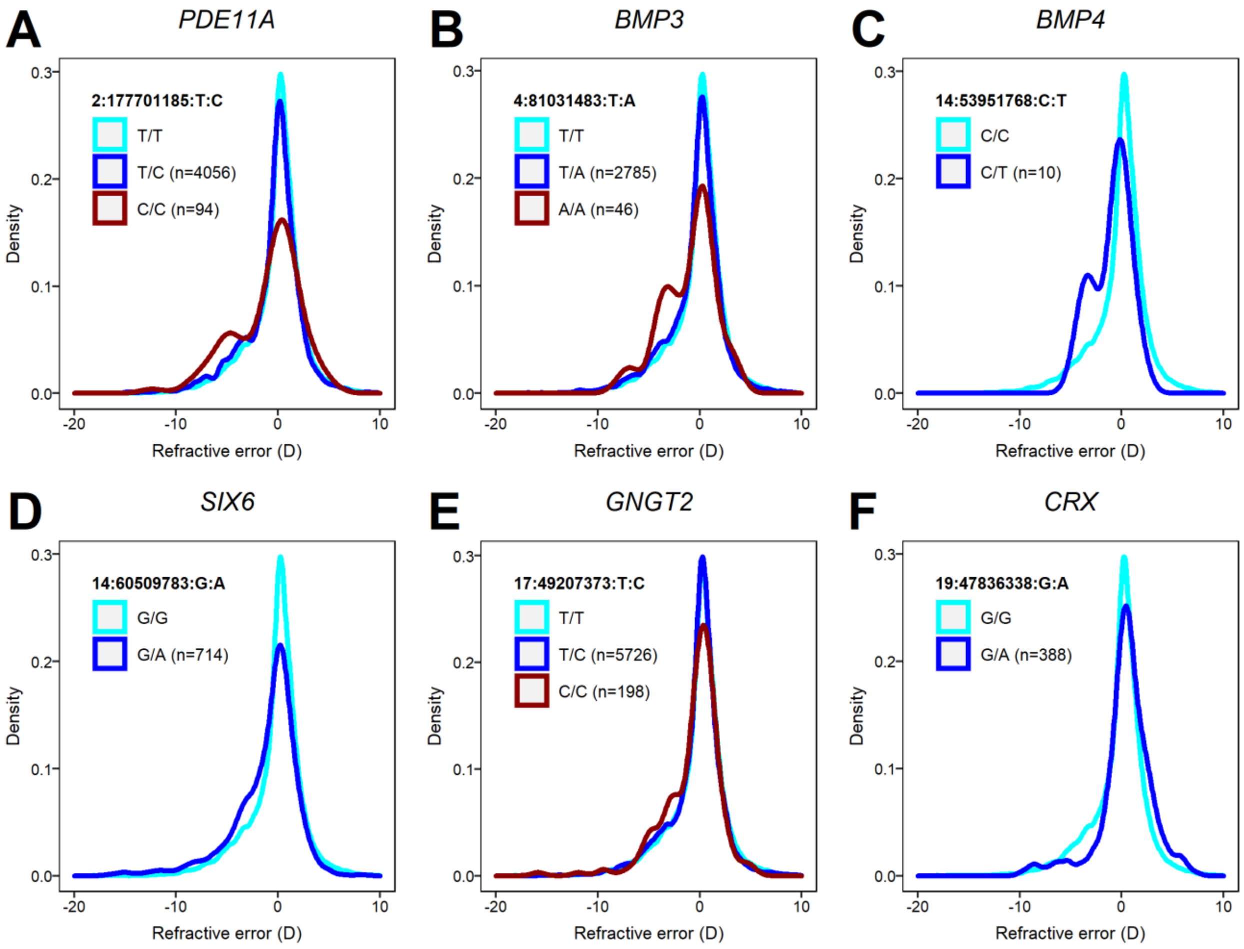

Figure S4. Fine-mapping and conditional analysis of the *BMP4* gene region. Panel **A** displays the results from fine-mapping analysis with *SUSIE*. The subpanels show the evidence for association with refractive error (‘Z-score’), the statistical confidence that a variant is a putative causal variant (‘PIP’; probability units on scale 0–1), and the relative effect size of the variant (‘BHAT’; units of standard deviation change in refractive error per copy of risk allele). Independent putative causal variants are shaded in different colours. Panels **B** and **C** display the results of the conditional GWAS analyses. Subpanels show GWAS results before (‘Original’) and after (‘Conditional’) conditioning on the specified lead variant in the region. The lead variant is depicted as a green diamond. Note that rare variant 14:53951768:C:T was not amongst the variants included in the original GWAS and that conditioning on this variant did not appreciably impact on the GWAS regional association plots (panel C).

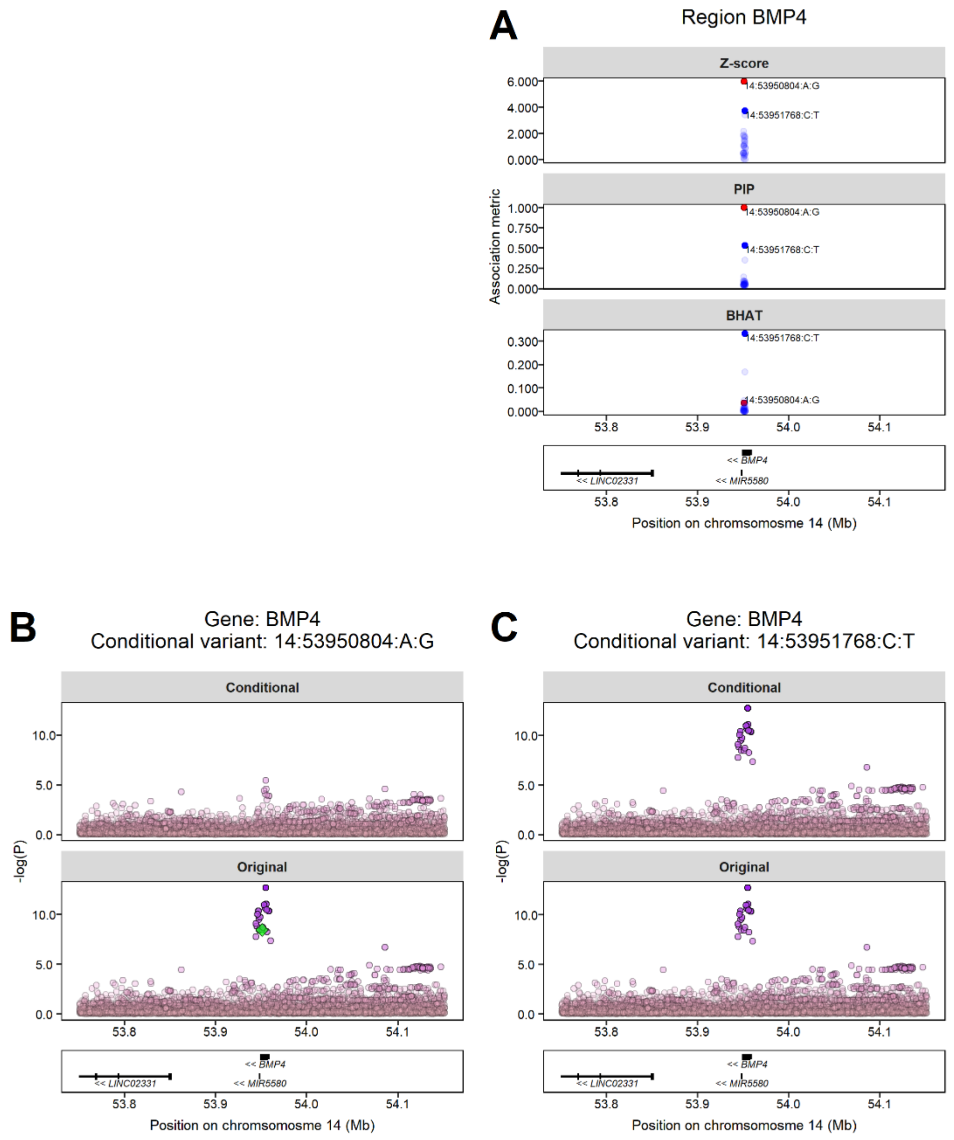

Figure S5. Fine-mapping and conditional analysis of the *SIX6* and *CRX* gene regions. Panels **A** and **B** display the results from fine-mapping analysis. Panels **C**, **D** and **E** display the results of the conditional GWAS analyses. See Figure S4 for details of subpanels.

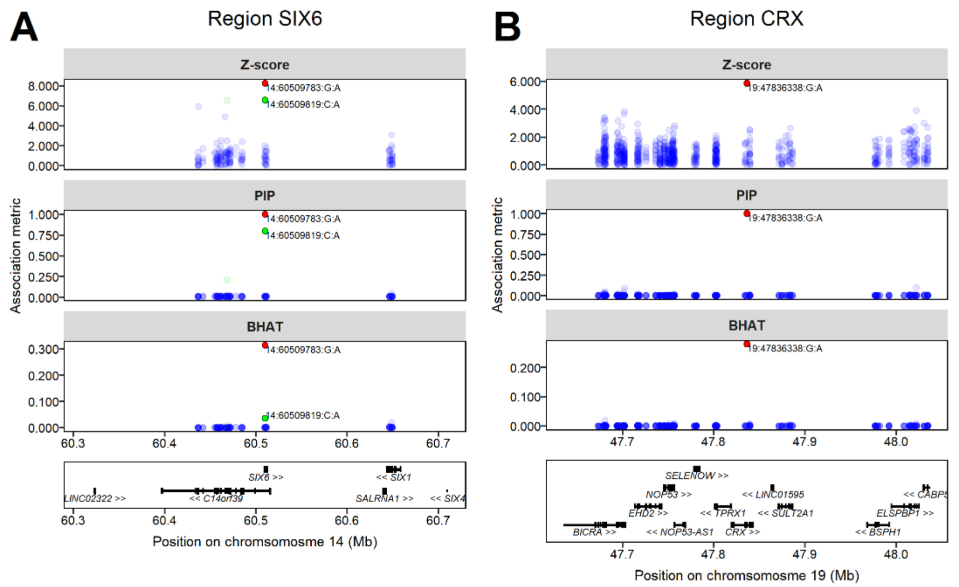

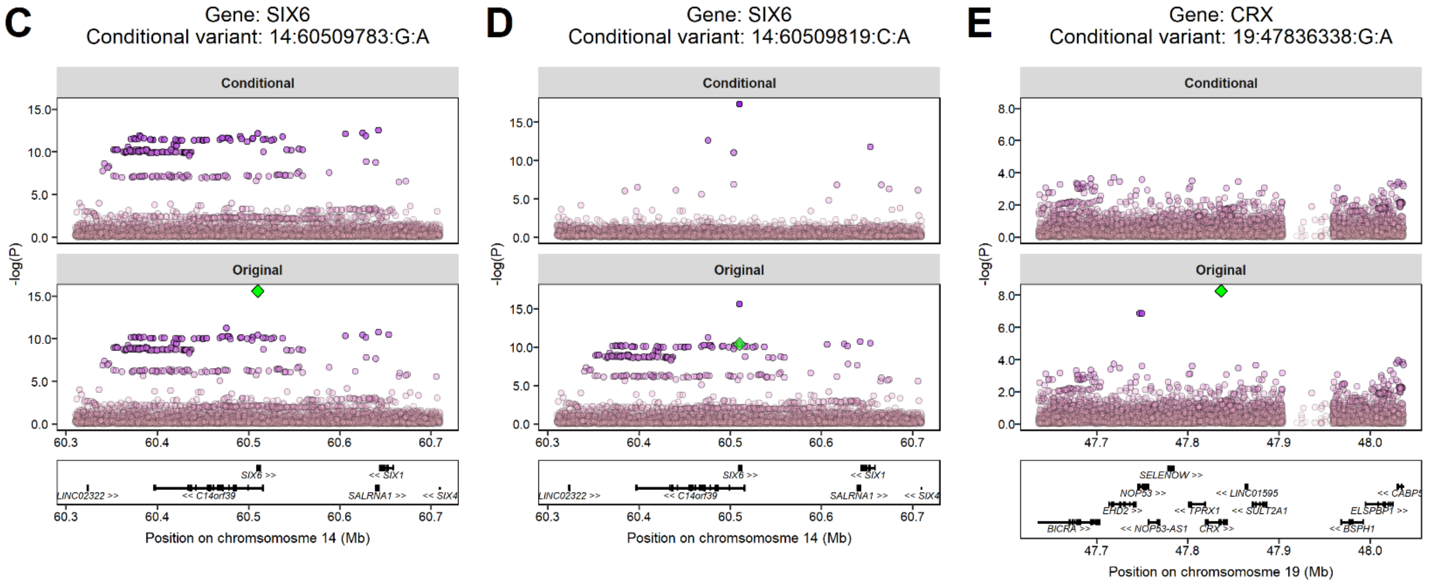

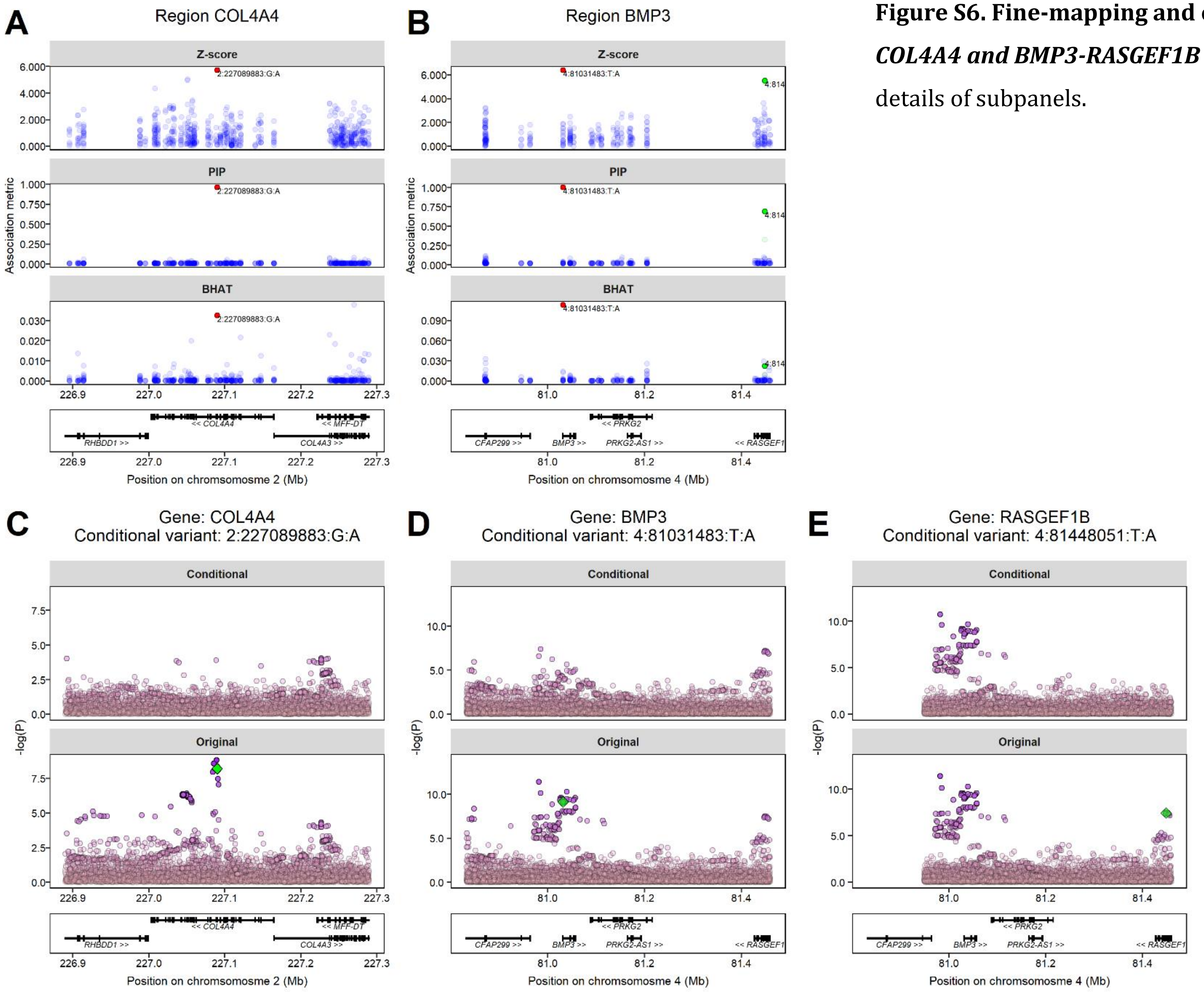

Figure S6. Fine-mapping and conditional analysis of the *COL4A4 and BMP3-RASGEF1B* gene regions. See Figure S4 for details of subpanels.

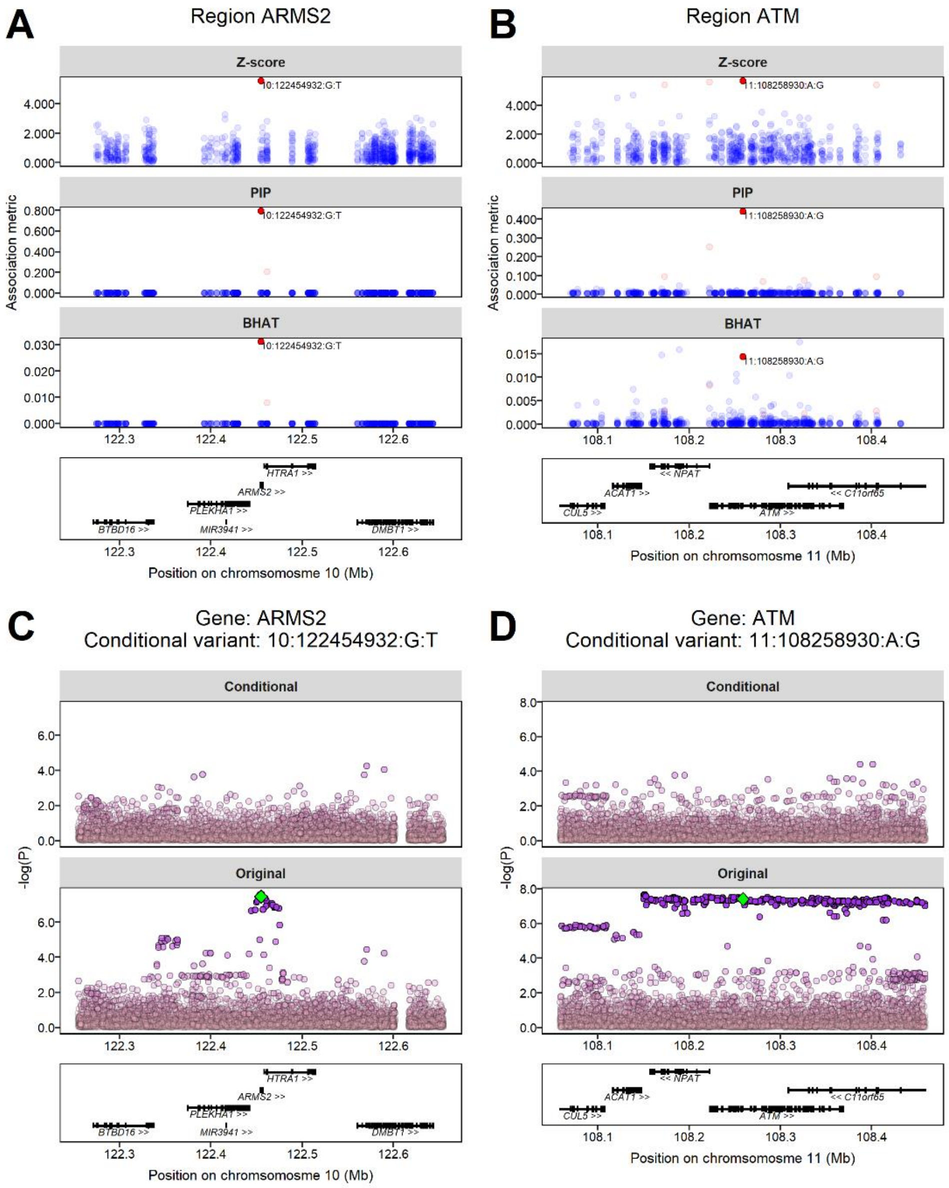

Figure S7.

Fine-mapping and conditional analysis: *ARMS2 and NPAT-ATM.* See Figure S4 for details of subpanels.

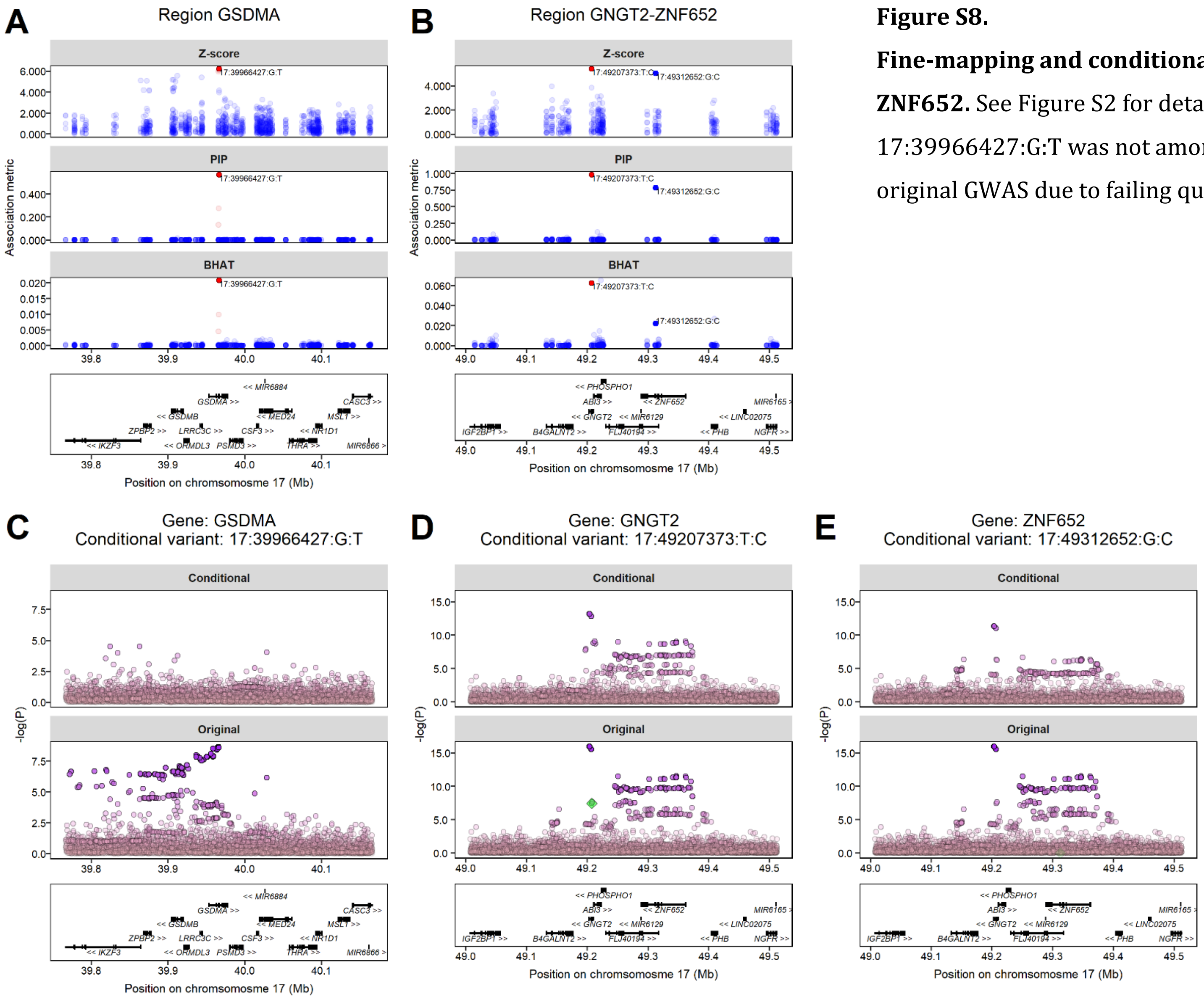

Figure S8.

Fine-mapping and conditional analysis: *GSDMA and GNGT2-ZNF652*. See Figure S2 for details of subpanels. Note that variant 17:39966427:G:T was not amongst the variants included in the original GWAS due to failing quality control.

Figure S9.

Fine-mapping and conditional analysis: *PDE11A* and *RGR*. See Figure S2 for details of subpanels.

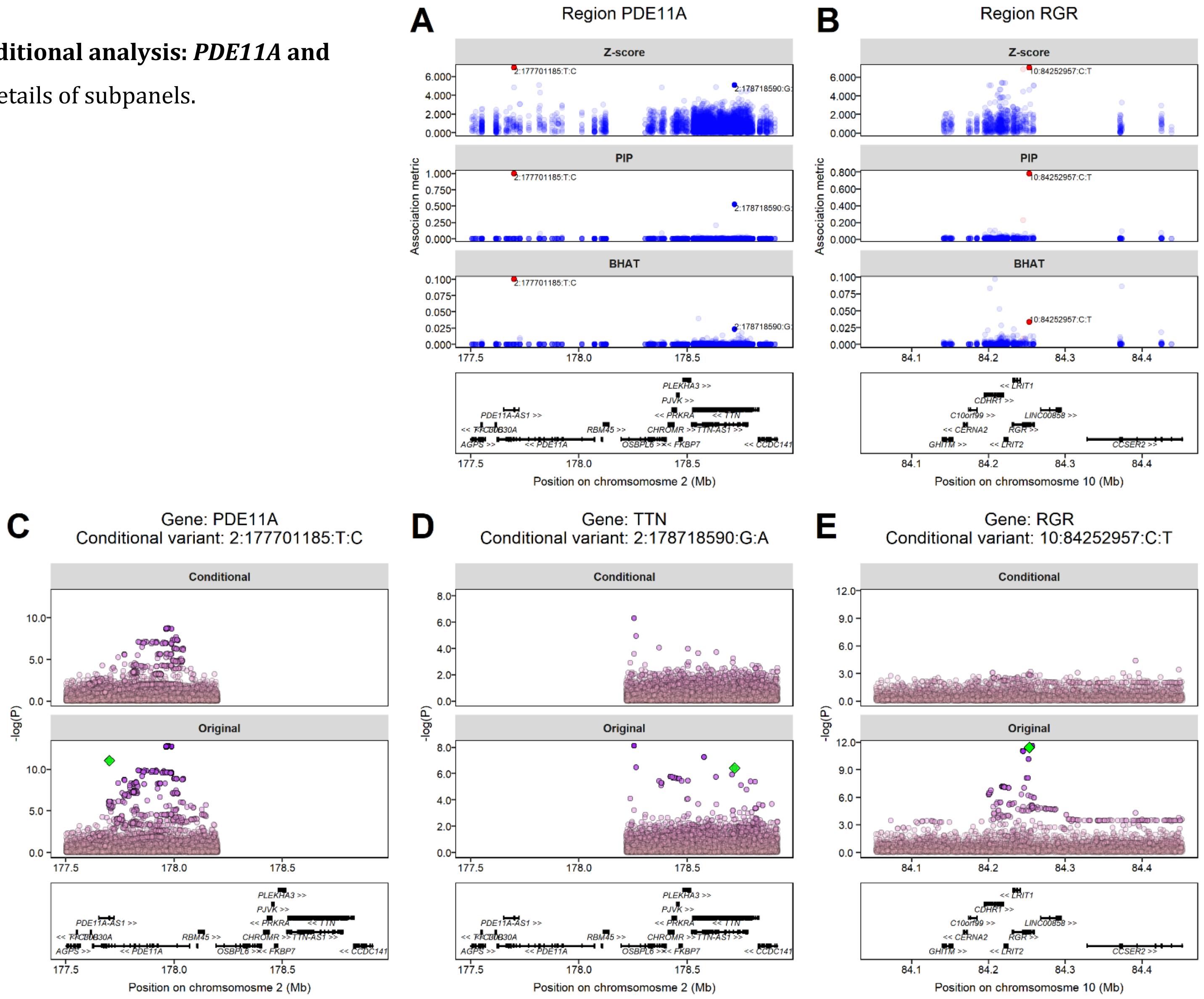

Figure S10.

Fine-mapping and conditional analysis: *PRSS56 and KAZALD1*. See Figure S2 for details of subpanels.

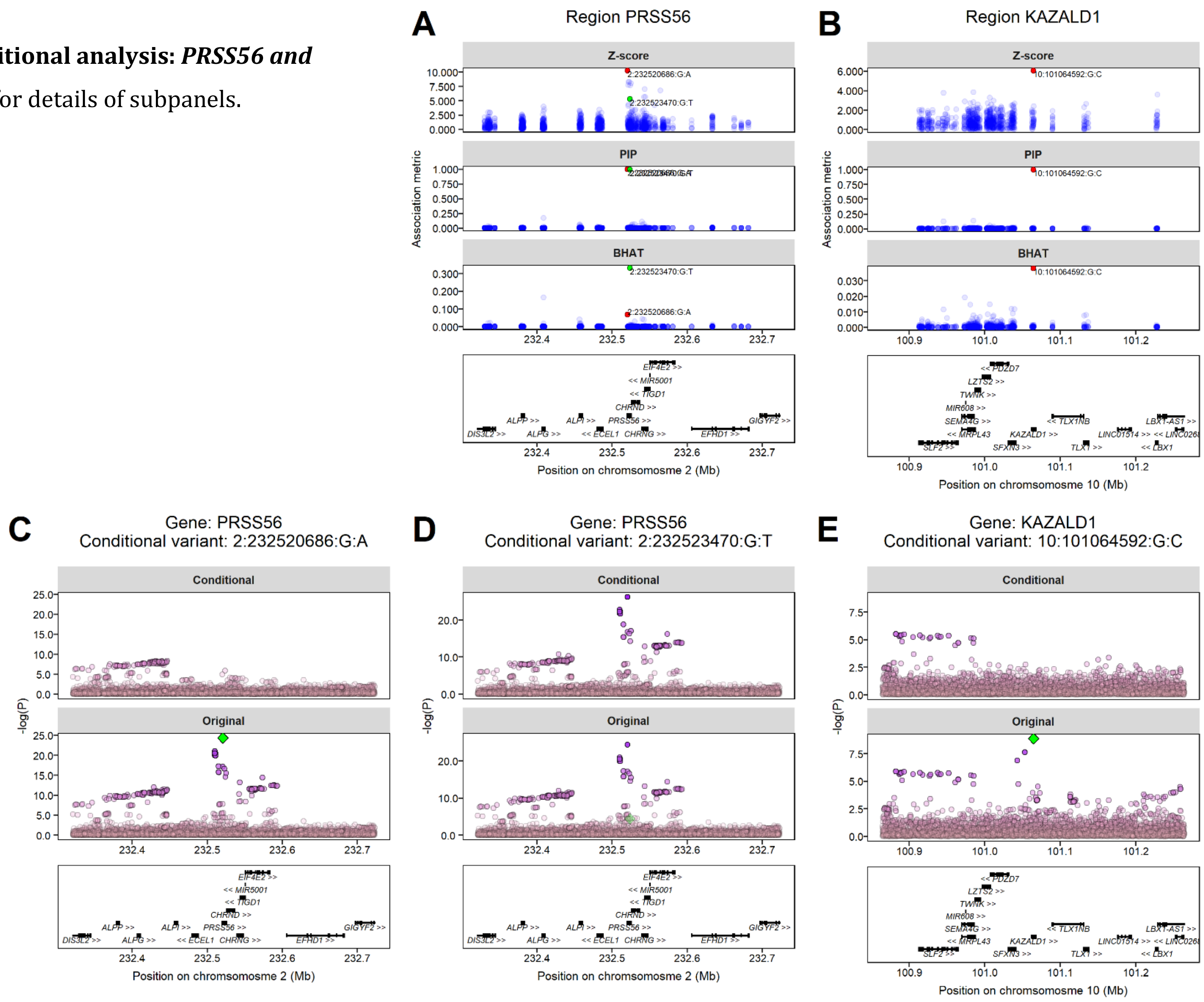

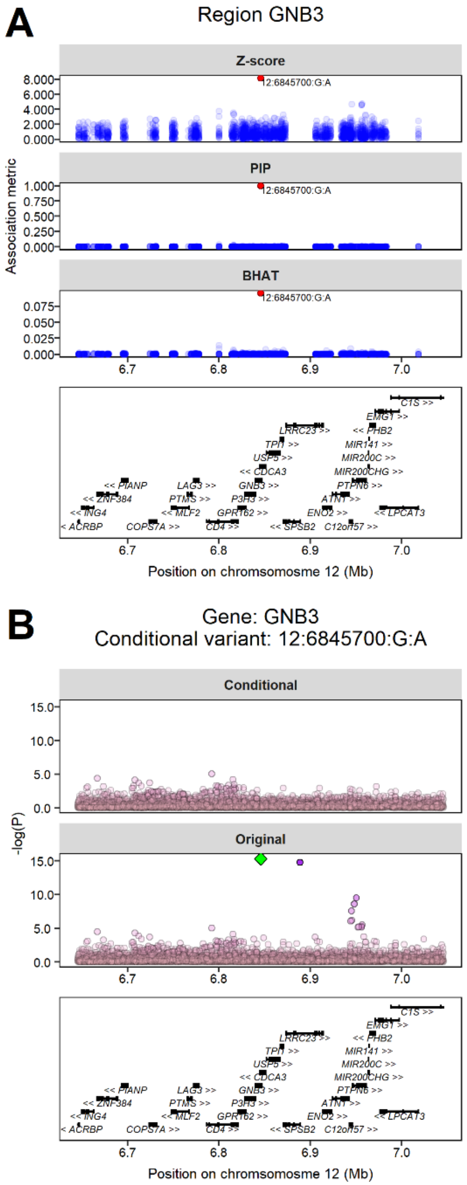

Figure S11.

Fine-mapping and conditional analysis: *GNB3*.

See Figure S2 for details of subpanels.

Figure S12. Power to detect a genetic variant associated with refractive error as a function of effect size and minor allele count (MAC). Error bars show 95% confidence intervals.

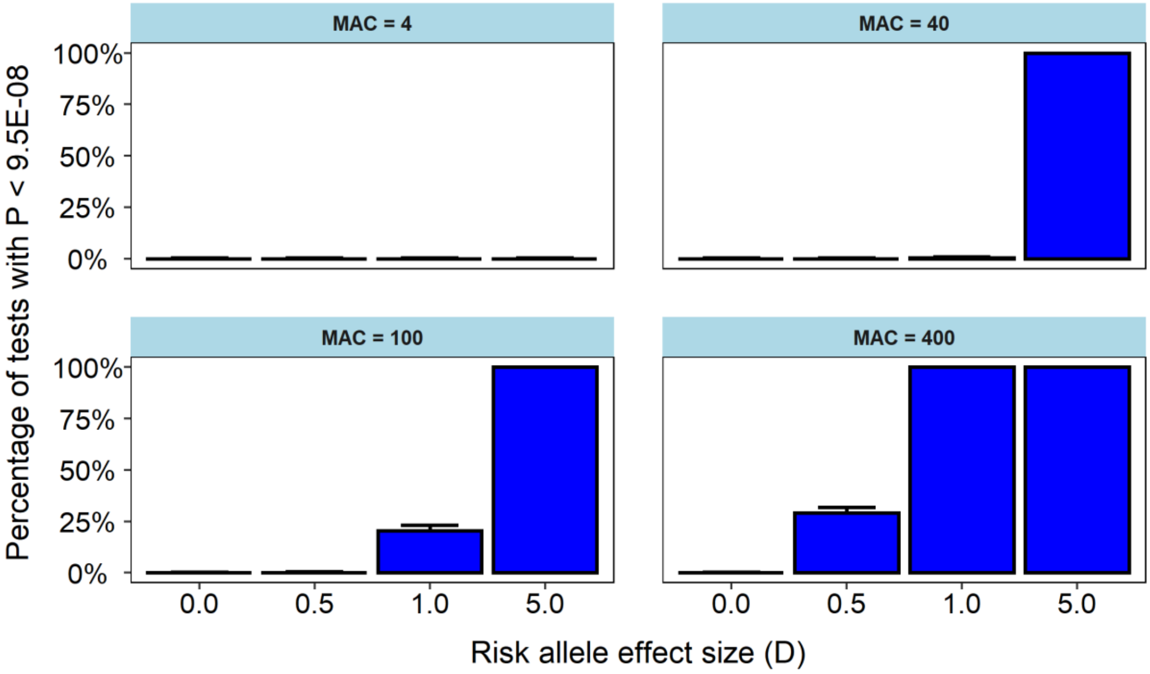

Supplementary References

1 Pozarickij, A., Williams, C., Hysi, P.G., Guggenheim, J.A. and U. K. Biobank Eye and Vision Consortium. (2019) Quantile regression analysis reveals widespread evidence for gene-environment or gene-gene interactions in myopia development. *Commun. Biol.*, **2**, 167.

2 Cingolani, P., Platts, A., Wang, L.L., Coon, M., Nguyen, T., Wang, L., Land, S.J., Lu, X. and Ruden, D.M. (2012) A program for annotating and predicting the effects of single nucleotide polymorphisms, SnpEff. *Fly*, **6**, 80-92.

3 Van Hout, C.V., Tachmazidou, I., Backman, J.D., Hoffman, J.D., Liu, D., Pandey, A.K., Gonzaga-Jauregui, C., Khalid, S., Ye, B., Banerjee, N. *et al.* (2020) Exome sequencing and characterization of 49,960 individuals in the UK Biobank. *Nature*, **586**, 749-756.

4 Chang, C.C., Chow, C.C., Tellier, L.C., Vattikuti, S., Purcell, S.M. and Lee, J.J. (2015) Second-generation PLINK: rising to the challenge of larger and richer datasets. *GigaScience*, **4**, 7.

5 Wang, G., Sarkar, A., Carbonetto, P. and Stephens, M. (2019) A simple new approach to variable selection in regression, with application to genetic fine-mapping. *bioRxiv*, in press., 501114.

6 Kiefer, A.K., Tung, J.Y., Do, C.B., Hinds, D.A., Mountain, J.L., Francke, U. and Eriksson, N. (2013) Genome-wide analysis points to roles for extracellular matrix remodeling, the visual cycle, and neuronal development in myopia. *PLoS Genet.*, **9**, e1003299.

7 Orr, A., Dube, M.-P., Zenteno, J.C., Jiang, H., Asselin, G., Evans, S.C., Caqueret, A., Lakosha, H., Letourneau, L., Marcadier, J. *et al.* (2011) Mutations in a novel serine protease PRSS56 in families with nanophthalmos. *Mol. Vision*, **17**, 1850-1861.

8 Vishweswaraiah, S., Swierkowska, J., Ratnamala, U., Mishra, N.K., Guda, C., Chettiar, S.S., Johar, K.R., Mrugacz, M., Karolak, J.A., Gajecka, M. *et al.* (2019) Epigenetically dysregulated genes and pathways implicated in the pathogenesis of non-syndromic high myopia. *Scientific Reports*, **9**, 4145.

9 Tedja, M.S., Wojciechowski, R., Hysi, P.G., Eriksson, N., Furlotte, N.A., Verhoeven, V.J.M., Iglesias, A.I., Meester-Smoor, M.A., Tompson, S.W., Fan, Q. *et al.* (2018) Genome-wide association meta-analysis highlights light-induced signaling as a driver for refractive error. *Nat. Genet.*, **50**, 834–848.

10 Hysi, P.G., Choquet, H., Khawaja, A.P., Wojciechowski, R., Tedja, M.S., Yin, J., Simcoe, M.J., Patasova, K., Mahroo, O.A., Thai, K.K. *et al.* (2020) Meta-analysis of 542,934 subjects of European ancestry identifies new genes and mechanisms predisposing to refractive error and myopia. *Nat. Genet.*, **52**, 401-407.
